# Lipid Pathway Alterations in Parkinson's Disease Primary Visual Cortex

**DOI:** 10.1371/journal.pone.0017299

**Published:** 2011-02-28

**Authors:** Danni Cheng, Andrew M. Jenner, Guanghou Shui, Wei Fun Cheong, Todd W. Mitchell, Jessica R. Nealon, Woojin S. Kim, Heather McCann, Markus R. Wenk, Glenda M. Halliday, Brett Garner

**Affiliations:** 1 Neuroscience Research Australia, Sydney, New South Wales, Australia; 2 Department of Biochemistry, National University of Singapore, Singapore, Singapore; 3 Life Science Institute, National University of Singapore, Singapore, Singapore; 4 School of Health Sciences, University of Wollongong, Wollongong, New South Wales, Australia; 5 Illawarra Health and Medical Research Institute, University of Wollongong, Wollongong, New South Wales, Australia; 6 School of Chemistry, University of Wollongong, Wollongong, New South Wales, Australia; 7 School of Medical Sciences, University of New South Wales, Sydney, New South Wales, Australia; 8 Department of Biological Sciences, National University of Singapore, Singapore, Singapore; 9 School of Biological Sciences, University of Wollongong, Wollongong, New South Wales, Australia; Federal University of Rio de Janeiro, Brazil

## Abstract

**Background:**

We present a lipidomics analysis of human Parkinson's disease tissues. We have focused on the primary visual cortex, a region that is devoid of pathological changes and Lewy bodies; and two additional regions, the amygdala and anterior cingulate cortex which contain Lewy bodies at different disease stages but do not have as severe degeneration as the substantia nigra.

**Methodology/Principal Findings:**

Using liquid chromatography mass spectrometry lipidomics techniques for an initial screen of 200 lipid species, significant changes in 79 sphingolipid, glycerophospholipid and cholesterol species were detected in the visual cortex of Parkinson's disease patients (n = 10) compared to controls (n = 10) as assessed by two-sided unpaired *t*-test (*p-*value <0.05). False discovery rate analysis confirmed that 73 of these 79 lipid species were significantly changed in the visual cortex (*q*-value <0.05). By contrast, changes in 17 and 12 lipid species were identified in the Parkinson's disease amygdala and anterior cingulate cortex, respectively, compared to controls; none of which remained significant after false discovery rate analysis. Using gas chromatography mass spectrometry techniques, 6 out of 7 oxysterols analysed from both non-enzymatic and enzymatic pathways were also selectively increased in the Parkinson's disease visual cortex. Many of these changes in visual cortex lipids were correlated with relevant changes in the expression of genes involved in lipid metabolism and an oxidative stress response as determined by quantitative polymerase chain reaction techniques.

**Conclusions/Significance:**

The data indicate that changes in lipid metabolism occur in the Parkinson's disease visual cortex in the absence of obvious pathology. This suggests that normalization of lipid metabolism and/or oxidative stress status in the visual cortex may represent a novel route for treatment of non-motor symptoms, such as visual hallucinations, that are experienced by a majority of Parkinson's disease patients.

## Introduction

Parkinson's disease (PD) is an idiopathic neurodegenerative movement disorder with a prevalence of approximately 1–2% of the population over 65 years increasing to 3–5% in people over 85 years old [1], [2]. Pathologically, PD classically presents with specific loss of dopaminergic neurons in the substantia nigra pars compacta (SN) and Lewy body formation [3]. Lewy bodies are composed of aggregated proteins including alpha-synuclein (α-syn) and other components including lipids [4], [5]. According to the Braak PD staging scheme, selective structures of the brainstem, temporal mesocortex and neocortex become progressively involved over time, with severe destruction of the SN and involvement of the amygdala (AMY) by stage 4, and the anterior cingulate cortex (ACC) affected by stage 5 [6], [7]. The occipital cortex is pathologically spared in PD [3], [6], [8]. The attractiveness of the Braak staging scheme is that it explains many of non-motor symptoms in PD [8], although there is controversy over this concept [9], [10].

Non-motor clinical symptoms of PD refer to a combination of sleep disturbances, autonomic dysfunction, sensory complications, olfactory deficits, and neuropsychiatric problems. Of these neuropsychiatric symptoms, visual hallucinations (VH) are one of the most common. In a recent 20 year follow-up study, VH were present at a prevalence of 74% in idiopathic PD patients [11]. Other studies of shorter follow-up duration have indicated VH prevalence in the range of ∼50 to 75% [12]–[15]. Although VH were initially suggested to be a complication of treatment, it is now thought that changes in neural circuits underlie the dysfunction of visual pathways [16]. Structural MRI studies have shown that PD patients with VH present with grey matter atrophy of the occipito-parietal and hippocampal regions of the brain [17]–[19]. In addition to this, PET and functional MRI studies have demonstrated reduced activation of the ventral/lateral visual association cortices and the primary visual cortex (VC) in particular [20]–[23]. To date, there is no evidence of pathology in the VC of PD patients [7], even though dysfunction of this region is associated with VH. This implies that metabolic changes in the VC could contribute to the dysfunction of visual perception in PD.

Maintenance of lipid homeostasis is increasingly recognized as a crucial factor for normal neuronal function. There are several reasons to suspect that modulation of cerebral lipid metabolism or transport may be linked to PD. These include the findings that α-syn is a lipid binding protein and that it deposits with lipids associated with Lewy bodies and neuromelanin in PD tissues [4], [5], [24]. Genetic deletion of α-syn in mice results in increased levels of cerebral cholesterol, cholesteryl esters and triacylglycerols [25], whereas changes in multiple classes of phospholipids were detected in old (but not young) transgenic mice expressing human α-syn [26]. Recent studies also suggest that the association of α-syn with oxidized lipid metabolites can lead to mitochondrial dysfunction in PD [27]. Other studies have suggested changes in cerebral cholesterol, oxysterols, and cholesterol hydroperoxides may be related to PD progression [28]–[30] and it has been established that mutations in the GBA gene, that encodes glucocerebrosidase, confer increased risk for PD [31], [32]. Taken together, these studies suggest that changes in cerebral lipid homeostasis may contribute to neurodegenerative pathways in PD and possibly also to deficits in the VC that currently have no known pathological basis.

Lipidomics is as a powerful research tool that can be utilised to investigate lipid pathways that play important roles in cell biology and in specific disease processes. Lipidomics approaches have therefore been used to investigate lipid metabolism at the cellular level, in animal studies and increasingly in the human pathophysiological context [33]–[42]. In the present study we have undertaken the first lipidomics analysis of human PD tissues. We have focused on the primary VC and two additional brain regions, the AMY and ACC which contain Lewy bodies at different disease stages but do not have as severe degeneration as the SN at end-stage PD. The lipidomics data was confirmed by follow-up mass spectrometry and high-performance liquid chromatography (HPLC) techniques that were used to inform a targeted assessment of lipid pathway gene expression. Our data reveal substantial changes in sphingolipid and glycerophospholipid biosynthetic pathways in the VC of PD patients compared to controls. Levels of oxysterols derived from both non-enzymatic (free radical-mediated) and enzymatic pathways were also increased in the PD VC. Many of these changes in VC lipids were correlated with relevant changes in the expression of genes involved in lipid metabolism and an oxidative stress response.

## Materials and Methods

### Ethics statement

This research was conducted according to the principles expressed in the Declaration of Helsinki. Ethics approval was from the University of New South Wales Human Research Ethics Committee.

### Materials

All organic solvents used were of analytical/HPLC grade and purchased from Merck (Darmsdadt, Germany). Standard solutions of oxysterols, 5-alpha cholestane, and other cholesterol biosynthetic precursors were diluted in ethanol. Formic acid, acetic acid (Lancaster, England), potassium hydroxide, butylated hydroxytoluene (BHT), ethanol, acetic acid (Merck, Darmstadt, Germany), and hexane (Tedia, OH, USA) were of analytical grade. Methanol (EM Science, Darmstadt, Germany) and ethyl acetate (Fisher Scientific, UK) were of HPLC grade. Oasis mixed anion-exchange cartridges were from Waters Corp. (Milford, MA, USA). Recombinant human α-syn protein used as a Western blot standard was generously provided by Wei Ping Gai, Flinders University, Adelaide, Australia.

### Human Brain Tissue

Frozen grey matter brain tissue from 10 sporadic PD cases and 10 control cases was received from the Sydney Brain Bank and the NSW Tissue Resource Centre, part of the Australian Brain Bank Network funded by the National Health and Medical Research Council of Australia. Standardized clinicopathological criteria were used for diagnosis [43]. The demographic and basic clinical and neuropathological details for all cases and controls are provided in Table 1.

**Table 1:** Demographic and limited clinical and neuropathological brain donor details.

Case #	Age at death (y)	Gender (M/F)	PD duration (y)	Visual hallucinations (Y/N)	Post- mortem interval (h)	Braak PD stage (0-VI)	Braak neuritic stage (0-VI)
Con 1	93	F	-	N	21	0	0
Con 2	83	F	-	N	7	0	0
Con 3	79	M	-	N	8	0	0
Con 4	102	F	-	N	5	0	0
Con 5	92	F	-	N	16	0	0
Con 6	86	M	-	N	15	0	I
Con 7	85	M	-	N	9	0	I
Con 8	88	M	-	N	9	0	II
Con 9	87	F	-	N	5	0	0
Con 10	85	F	-	N	10	0	II
PD 1	78	M	24	Y	6	V	0
PD 2	84	M	17	N	7	IV	0
PD 3	66	M	12	Y	6	V	0
PD 4	91	F	10	Y	4	IV	III
PD 5	83	F	14	Y	32	V	III
PD 6	90	M	15	N	5	V	0
PD 7	72	M	9	N	4	IV	0
PD 8	83	F	14	Y	7	V	0
PD 9	75	M	14	Y	9	V	II
PD 10	69	M	17	Y	5	V	I

The PD cases had a mean age of 79±9 y, a mean disease duration of 15±4 y and a mean postmortem interval of 8.5±8 h. The controls were 9 years older on average (mean age of 88±6 y, *t*-test *p* = 0.02) and had a similar postmortem interval (mean of 10.5±5 h, *t*-test *p* = 0.5). Age was factored into the analyses as described in the Results section. There was no difference in the sex distribution between groups (χ^2^ *p* = 0.37) and both groups had similar causes of death; which included pneumonia (2 PD, 2 controls), terminal prostate cancer (1 PD, 1 control), sepsis/renal failure (1 PD, 1 control) and cardiac events or cardiorespiratory arrest (remaining PD and controls).

Approximately 500 mg of frozen brain tissue from the AMY, ACC and VC was pulverized over dry ice and four aliquots of approximately 10 mg, 50 mg, 100 mg, 100 mg of accurately weighed pulverised tissue samples were frozen and stored at −80°C until required for analysis by Western blotting, mass spectrometry, HPLC or quantitative real-time (qRT-PCR). These methods are briefly summarised below and a full description is included as Methods S1. A schematic diagram outlining the workflow of the complete lipidomic screening strategy is also included as Figure S1.

### Western blotting for α-syn and synaptophysin

The brain tissue was homogenized into three fractions as described previously [44], [45] to provide homogenates that were soluble in tris-buffered saline (TBS), TBS containing 1% (w/v) Triton X-100 (TX) and an SDS-solublized pellet fraction (SDS). Equal amounts of protein were then analysed by SDS-PAGE and Western blotting using α-syn or synaptophysin monoclonal antibodies and re-probed with a rabbit β-actin polyclonal antibody. Signal intensities were quantified using NIH Image J software (National Institutes of Health, Bethesda, MD) with the relative expression of bands of interest normalised to β-actin. Full details are included as Methods S1.

### Analysis of lipids using high performance liquid chromatography/mass spectrometry (LC/MS) and gas chromatography/mass spectrometry (GC/MS)

Lipids were extracted from tissue samples containing internal standards and heavy isotopes using a modified Bligh and Dyer extraction method [46]. The lipid extract was split into two aliquots for LC/MS and GC/MS. For the LC/MS analysis an Agilent high performance liquid chromatography (HPLC) 1200 system coupled with an Applied Biosystem Triple Quadrupole/Ion Trap mass spectrometer (3200 Qtrap) was used for quantification of individual phospholipids and sphingolipids [42], [47]. Neutral lipids were analyzed using a modified method from a previously described LC/MS method [48]. Free cholesterol was quantified using a HPLC atmospheric pressure chemical ionisation MS (LC/APCI/MS) method [49]. For the GC/MS analysis, aliquots of lipid extracts prepared as for LC/MS above were analysed using an Agilent 5975 inert XL mass selective detector and 5973 gas chromatograph equipped with an automatic sampler and a computer workstation. Full details are included as Methods S1.

### Electrospray ionisation MS

Samples were also analysed by direct ESI/MS. In brief, lipids were extracted using published methods [50], [51] and mass spectra were obtained using a Waters QuattroMicro™ (Waters, Manchester, U.K.) equipped with a z-spray electrospray ion source. Samples were infused into the electrospray ion source and sphingolipids and ceramides analysed as described previously [50], [52], [53]. Full details are included as Methods S1.

### High performance liquid chromatography

Cholesterol and α-tocopherol content of brain tissue was determined by reversed-phase HPLC using a C18 reversed phase column as described previously [54]. Trace amounts of [3H]-cholesterol were used as an internal standard. Full details are included as Methods S1.

### Quantitative real-time PCR

The qRT-PCR analysis of human brain samples was performed using our established methods [55]. Brain tissue was homogenized in TRIzol reagent (Invitrogen, Mount Waverly, Australia) and RNA concentration was determined spectrophometrically with a Nanodrop 1000 (Thermo scientific, Wilmington, DE). Five µg of total RNA was used for reverse transcription with random primers and M-MLV reverse transcriptase (Promega, Sydney, Australia). The resulting cDNA provided the template in the qRT-PCR, which was carried out using a Mastercycler EP Realplex S (Eppendorf, North Ryde, Australia). qRT-PCR of the house keeping gene, β-actin was also performed for each cDNA template and gene expression normalised to β-actin. RNA integrity was confirmed using a high resolution Bioanalyzer electrophoresis system (Agilent Technologies, Palo Alto, CA, USA) as described previously [55]. Full details are included as Methods S1. All primers were purchased from Sigma (Castle Hill, Australia) and details of the sequences are provided as Table S1.

### Statistics

Data presented are expressed as mean with SEM shown by the error bars. Statistical significance was analysed using the two-sided unpaired *t*-test and SPSS Statistics software (version 17, SPSS Inc. Chicago, IL). A *p-*value <0.05 was considered significant. For the lipidomics datasets, false discovery rate (FDR) *q*-values were calculated from the *t*-test *p-*values [56].

## Results

### Characterisation of α-syn and synaptophysin expression

**Figure 1:**
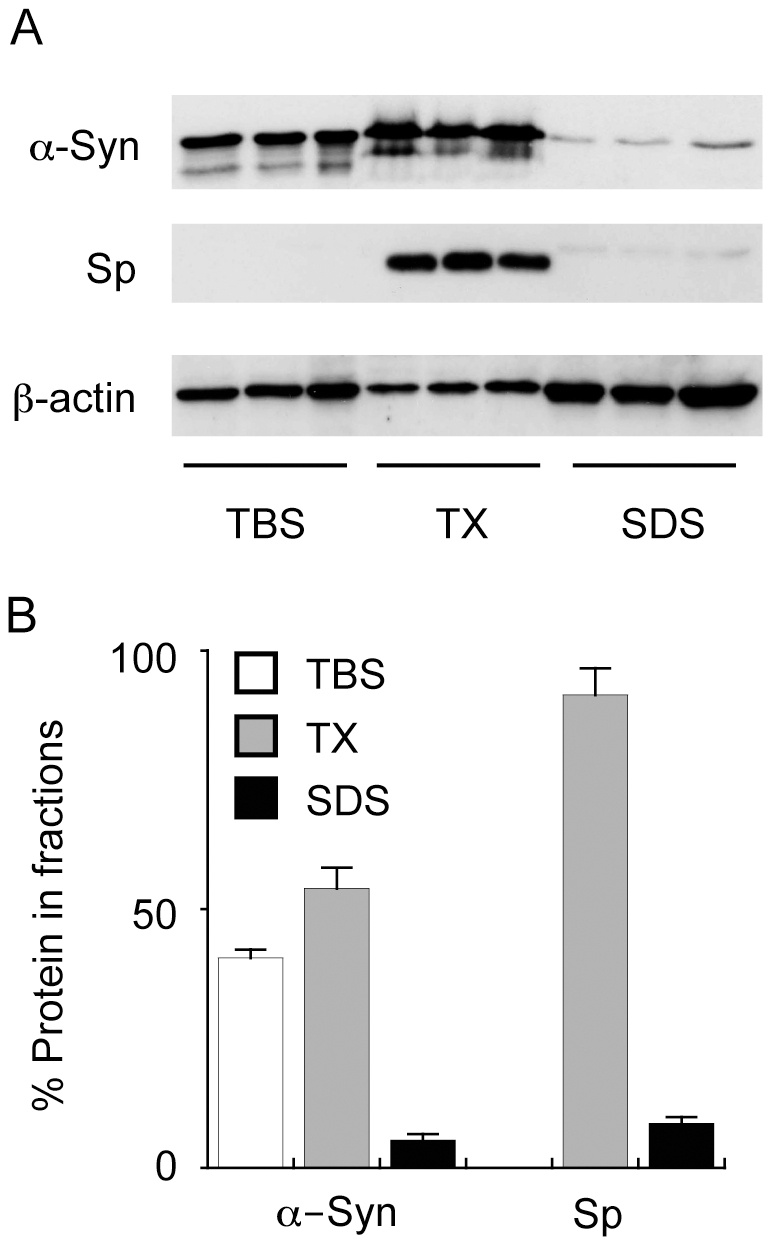
Analysis of α-synuclein and synaptophysin in fractionated Parkinson's disease tissues. Tissues were homogenised into three fractions that contained tris-buffered saline (TBS), TBS containing Triton X100 (TX) or sodium dodecyl sulphate (SDS) and α-synuclein (α-Syn), synaptophysin (Sp) and β-actin expression was analysed by Western blotting (A). The intensity of the bands was measured and the relative amounts of α-Syn and Sp in each fraction is expressed in the histogram (B). The data are derived from Parkinson's disease amygdala (PD AMY) samples and are used as an example to illustrate the techniques used to characterise the PD tissues. Data in “B” represent mean values with SEM shown by the error bars for the three samples shown in “A”.

The PD Braak staging for the cases and controls is given in Table 1. In order to provide a biochemical correlate of Lewy body pathology in the corresponding small tissue samples that we analysed, Western blotting for α-syn in SDS-soluble fractions of brain homogenate was performed [45]. As an example, AMY samples derived from PD cases are shown in Figure 1. α-Syn was detected predominantly in the TBS-soluble (41%) and TX-soluble (54%) fractions with a small but reproducibly detectable portion (5%) also detected in the SDS-soluble fraction (Fig. 1A,B). The amount of α-syn extracted in the SDS fraction reflects α-syn deposition in Lewy bodies [3]. In approximately 50% of PD cases, we also detected apparent high molecular weight (HMW) species of α-syn in the SDS fraction of the AMY (Fig. S2). A 31 kDa α-syn band was one of the clearest HMW bands detected; although a previous study has suggested this may be due to non-specific binding of the detection antibody [45]. These HMW species accounted for only a minor proportion of total α-syn and because they were not consistently observed, they were not quantified in the present study. Synaptophysin was also measured in the samples as a control for the fractionation method (as the membrane-bound synaptophysin should appear predominantly in the TX fraction) and as a surrogate marker for synaptic density/neuron loss. As predicted, the vast majority (91%) of synaptophysin was recovered in the TX fraction (Fig. 1A,B).

**Figure 2:**
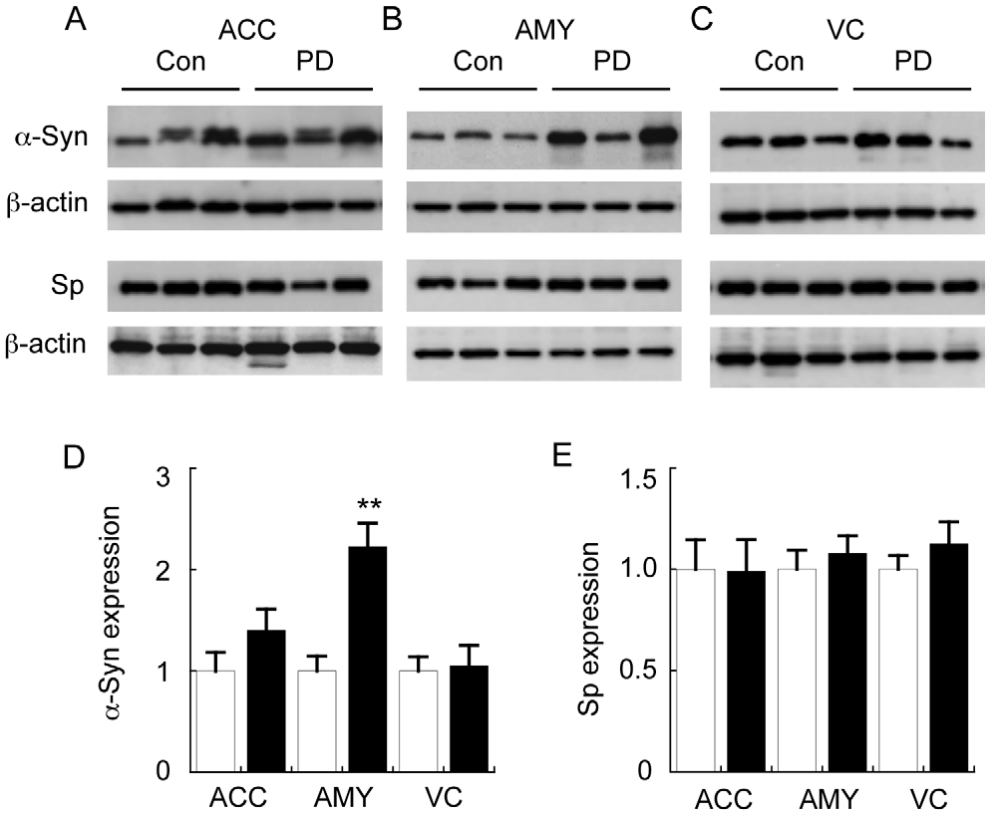
Analysis of control (Con) and Parkinson's disease (PD) α-synuclein (α-Syn) and synaptophysin (Sp) levels. Tissues were homogenised and fractionated as described in the legend to Figure 1 and insoluble α-Syn in the SDS fraction, and Sp in the TX-fraction was measured in the anterior cingulate cortex (ACC) (A), amygdala (AMY) (B), and visual cortex (VC) (C). Corresponding quantification of relative α-Syn (D) and Sp (E) protein expression in the three brain regions is provided in the histograms. Data represent mean ± SEM (n = 10), **p<0.001 by *t*-test.

The amounts of α-syn in the SDS-soluble fractions were then used to estimate relative Lewy body pathology in all samples. The data indicate that α-syn deposition was significantly increased in the SDS-soluble fractions of the AMY of the PD cases as compared to the controls (Fig. 2). A non-significant trend for increased α-syn deposition in the ACC was also noted whereas there were no changes in the VC (Fig. 2). This is consistent with the Braak stages for these cases with ACC Lewy bodies (Braak PD stage V) found in 7/10 of the PD cases (Table 1). Synaptophysin levels were not altered in any of the samples analysed, suggesting that extensive neurodegeneration or synaptic loss was not a feature of the brain regions analysed in this PD cohort (Fig. 2).

### LC/MS lipidomics assessment of control and PD tissues

**Figure 3:**
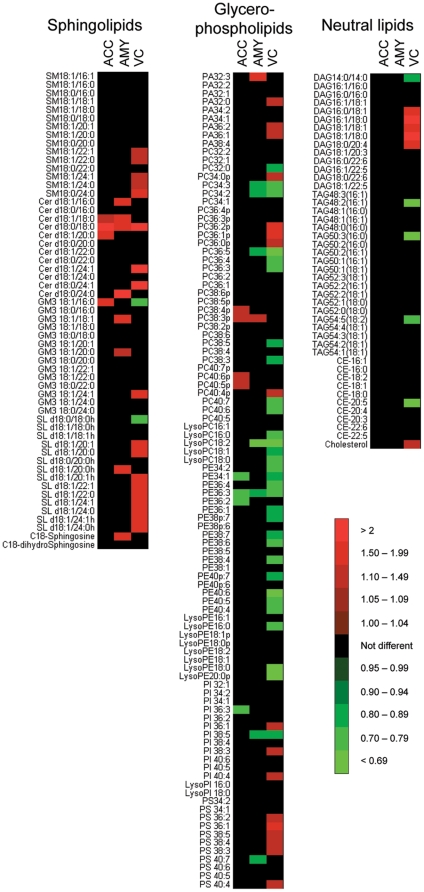
Heat map illustrating significant changes in lipid levels associated with Parkinson's disease as assessed using a lipidomics approach. Control (n = 10) and PD (n = 10) tissues were collected from the anterior cingulate cortex (ACC), amygdala (AMY) and visual cortex (VC). Lipids were extracted and analysed using an LC/MS lipidomics approach. The data indicates the fold change in the levels of sphingolipids, glycerophospholipids and neutral lipids detected in the PD samples relative to the Con samples. The intensity of the red and green colour represents the magnitude of increase or decrease, respectively, as indicated by the scale bar. Only statistically significant changes (*p*<0.05, *t*-test) are represented by the red and green colours in the heat map. Note: the nomenclature for phospholipid acyl chain length and saturation is abbreviated to improve clarity. The data used to generate the heat map is included as Tables S2 to S4.

A lipidomics analysis of ACC, AMY and VC tissues from both control and PD brains was then conducted. We initially focused on 200 lipid species that we categorised into three broad familes: *sphingolipids* (including C18-sphingosine, C18-dihydrosphingosine and multiple molecular species of sphingomyelin (SM), ceramide (Cer), ganglioside GM3 (GM3) and sulfatide (SL); *glycerophospholipids* (including multiple molecular species of phosphatidic acid (PA), phosphatidylcholine (PC), phosphatidylethanolamine (PE), phosphatidylinositol (PI) and phosphatidylserine (PS); and *neutral lipids* (including cholesterol and multiple molecular species of cholesteryl esters (CE), triacylglycerides (TAG) and diacylglycerides (DAG). In this analysis, each individual lipid in the PD samples was quantified relative to the levels detected in Con samples. The “heat map” of these lipid changes indicates significant changes in all brain areas examined (Fig. 3). Clearly, however, most of the statistically significant changes in lipid levels were detected in the VC (Fig. 3, Tables S2–S4). One issue that needs to be considered is the possibility that false discoveries are made due to the large number of test variables assessed using a lipidomics approach. We therefore used a robust method for estimating false discovery rates (FDR) that calculates *q*-values based on *p-*values derived from *t*-test analysis [56]. With a *q*-value threshold set at 5%, 73 of the 79 significant differences identified in the VC by the *t*-test remained statistically significant (Table S5). In contrast, all 17 and all 12 of the differences observed in the AMY and ACC were found to have *q*-values >0.05 (Table S5). This suggests that the changes detected in the VC are not due to chance whereas the changes detected in the AMY and ACC may be false positives and need to be interpreted with caution.

In the PD VC, the lipidomics data indicated an overall increase in sphingolipid levels, a decrease in PE accompanied by an increase in PS, and an overall increase in cholesterol and DAG (Fig. 3). To confirm the PD-related changes in lipid profiles identified by the lipidomics approach, independent quantitative lipid analysis methods were employed using the same Con (n = 10) and PD (n = 10) samples. To assess the possible influence of the 10% older age of the Con subjects (see Materials and Methods section), additional analyses were also performed that excluded the two oldest Con subjects and the two youngest PD subjects to provide age-matched groups (i.e. Con (n = 8) mean age 86.0±1.4 y, PD (n = 8) mean age 82.0±2.4 y (*t*-test, *p* = 0.2). The PD-related changes in lipid profiles were then used to inform a targeted assessment of changes in the expression of relevant genes involved in each of the lipid biosynthetic pathways.

### Changes in sphingolipid metabolism related to PD

**Figure 4:**
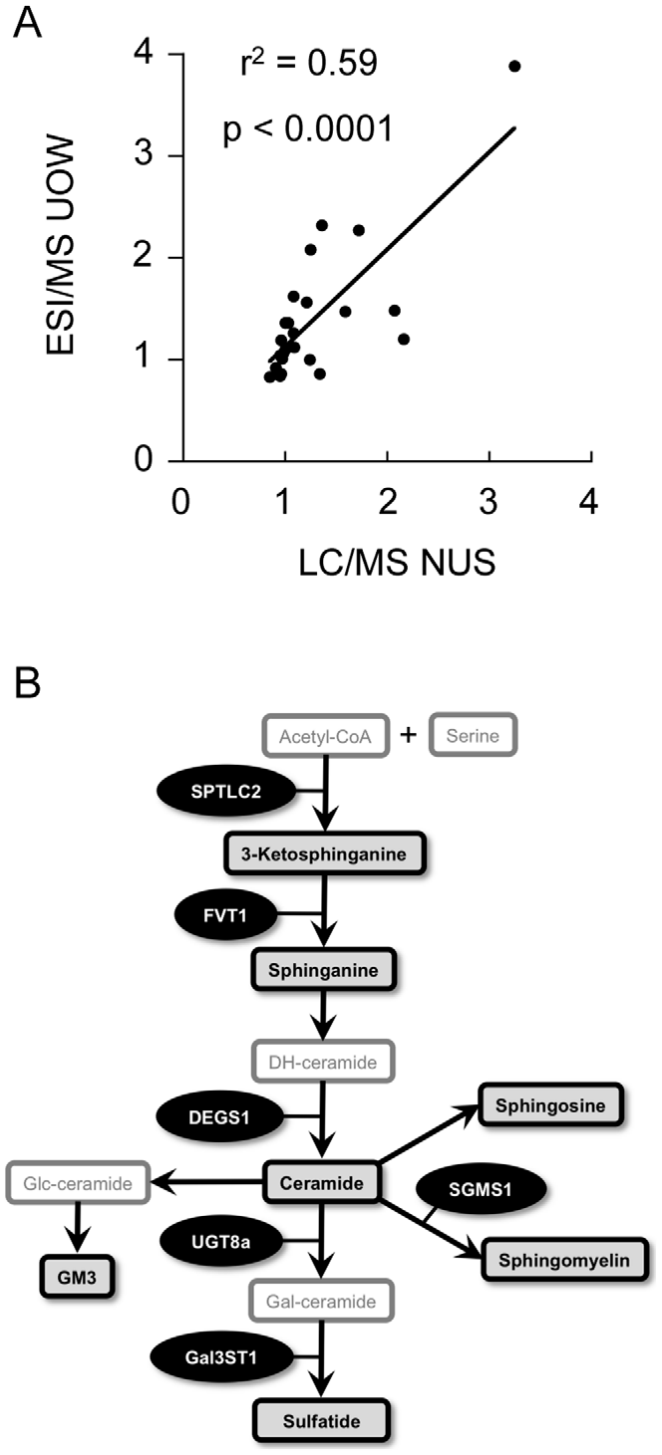
Comparison of liquid chromatography-mass spectrometry (LC/MS; National university of Singapore, NUS) and electrospray ionisation mass spectrometry (ESI/MS; University of Wollongong, UOW) techniques for the detection of sphingolipid changes in control (Con) and Parkinson's disease (PD) tissues. Independent aliquots of Con (n = 10) and PD (n = 10) tissues were collected from the anterior cingulate cortex, amygdala and visual cortex. Lipids were extracted and analysed using LC/MS lipidomics at NUS (LC/MS NUS) and ESI/MS at UOW (ESI/MS UOW). The fold-change in lipid levels of the PD samples relative to the controls is provided in the scatter plot that compares the data from the two laboratories (A). A simplified schematic diagram of relevant sphingolipids and related genes assessed in this study is provided (B). The lipids in the boxes with black borders were analysed in the present study. Serine palmitoyltransferase, long chain base subunit 2 (SPTLC2); follicular lymphoma variant translocation 1 (FVT1); degenerative spermatocyte homolog 1; lipid desaturase (DEGS1); sphingomyelin synthase 1 (SGMS1); UDP galactosyltransferase 8A (UGT8A); and galactose-3-O-sulfotransferase 1 (GAL3ST1). Pearson correlation analysis indicates a positive correlation between the independent analyses (*p*<0.0001).

To confirm the changes in sphingolipid metabolism identified by LC/MS lipidomics, separate aliquots of brain tissue were analysed by electrospray ionisation (ESI)/MS. The results for this analysis were significantly correlated (r^2^ = 0.59, p<0.0001) with the LC/MS lipidomics data (Fig. 4A, Table S6). Figure 4A indicates a degree of variation in this correlation that may be due to differences in the lipid extraction methods and MS analytical techniques employed (as summarized in Fig. S1). The ESI/MS analysis did however confirm that levels of sphingolipids (SM and Cer) were increased in the PD VC (Table S6 and Table S7). There were no significant correlations between age and either SM or Cer in the entire cohort (data not shown). The PD-related changes in SM and Cer were also detected in the smaller age-matched group comparison (n = 8, data not shown). This lack of impact of age was expected as previous data indicates that changes in these sphingolipids across the ages of the brain samples used in our study would be extremely small and thus undetectable [57], [58].

**Figure 5:**
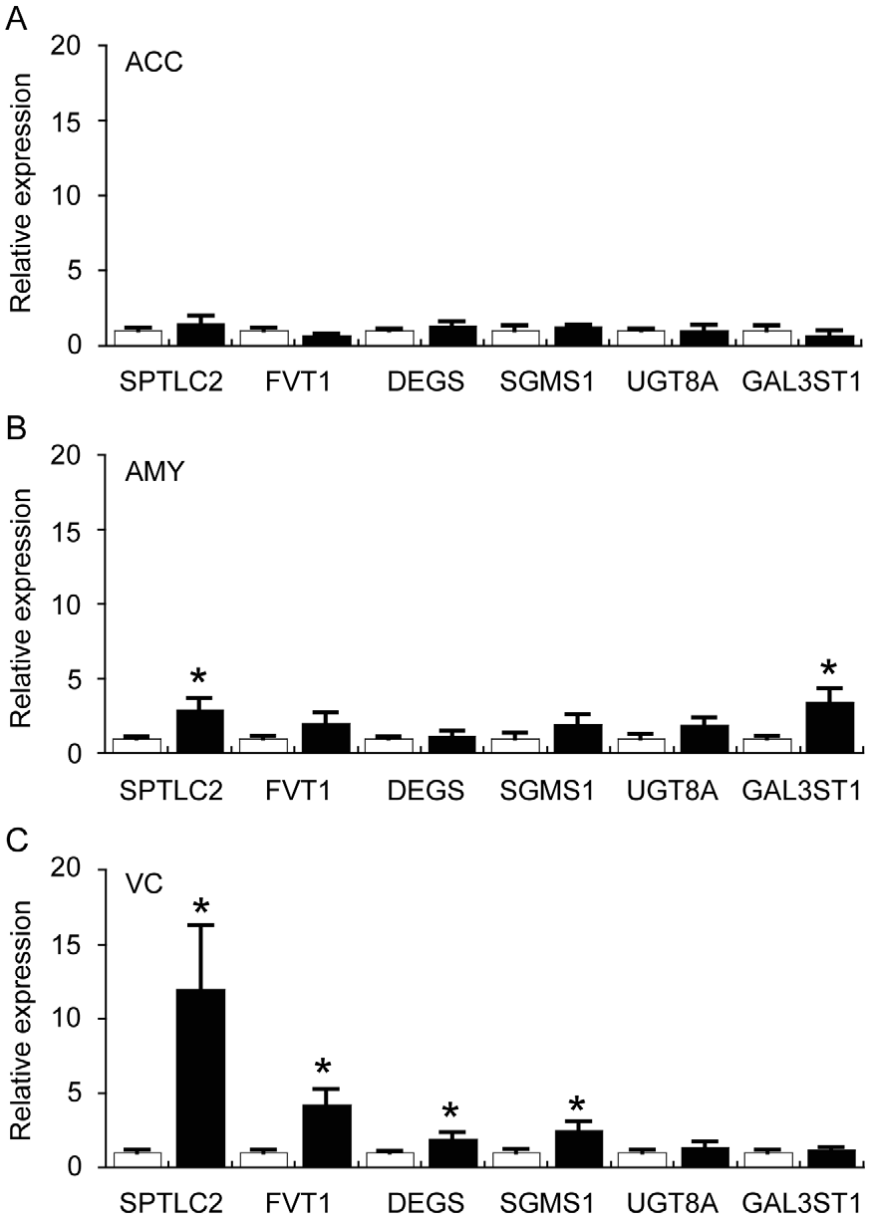
Quantitative real-time PCR analysis of selected sphingolipid-related genes in the anterior cingulate cortex (ACC), amygdala (AMY) and visual cortex (VC) of control (Con) and Parkinson's disease (PD) tissues. The expression of genes involved in the sphingolipid biosynthetic pathway (see Fig. 4B) was assessed by qRT-PCR. Data for all genes are expressed relative to the control values (Con  =  white bars, assigned a value of 1.0; PD  =  black bars). The data are presented separately for ACC (A), AMY (B) and VC (C). Serine palmitoyltransferase, long chain base subunit 2 (SPTLC2); follicular lymphoma variant translocation 1 (FVT1); degenerative spermatocyte homolog 1, lipid desaturase (DEGS); sphingomyelin synthase 1 (SGMS1); UDP galactosyltransferase 8A (UGT8A); and galactose-3-O-sulfotransferase 1 (GAL3ST1). Data represent mean ± SEM, *p<0.05 by *t*-test.

In order to understand the underlying mechanisms that may contribute to regional changes in sphingolipid metabolism in PD, we assessed the expression of a selection of sphingolipid pathway genes in the full sample cohort by qRT-PCR. A simplified scheme depicting the sphingolipid pathway with relevant lipids and genes we have focused on is shown in Figure 4B. The gene expression data indicated a significant up-regulation of several genes involved in Cer and SM synthesis (SPTLC2, FVT1, DEGS1, SGMS1) in the PD VC (Fig. 5). This is in general agreement with the lipidomics LC/MS and ESI/MS data and suggests that transcriptional activation contributes to the increased levels of Cer and SM detected in the PD VC. The increased levels of SL detected in the PD VC were not associated with changes in UGT8a or Gal3ST1; two genes that regulate the conversion of Cer to galactosylceramide (GalCer) and SL, respectively (Fig. 5). This may indicate that increased levels of SL detected in the PD VC are the result of decreased SL catabolism or that the level of expression of UGT8a/Gal3ST1 is sufficient to catalyse the conversion of a proportion of the increased Cer (substrate) observed to GalCer and SL.

### Changes in glycerophospholipid metabolism related to PD

We next focused on changes in glycerophospholipid metabolism that were revealed by the LC/MS lipidomics analysis. Although one of the glycerophospholipid molecular species (PC16:0/18:2) in one brain region (AMY) was found to be negatively correlated with age (Pearson correlation for whole group r^2^ = −0.34, *p* = 0.009, for smaller age-corrected group r^2^ = −0.58, *p* = 0.001), age did not contribute to PD-related differences in PC16:0/18:2 (i.e. rather than increased, the levels of this lipid were either unchanged or decreased in the PD tissues, Table S3). Previous detailed studies are consistent with only a very subtle decrease in glycerophospholipid levels with age [59]. The changes detected in PD are therefore not likely to be influenced by age in our analyses.

**Figure 6:**
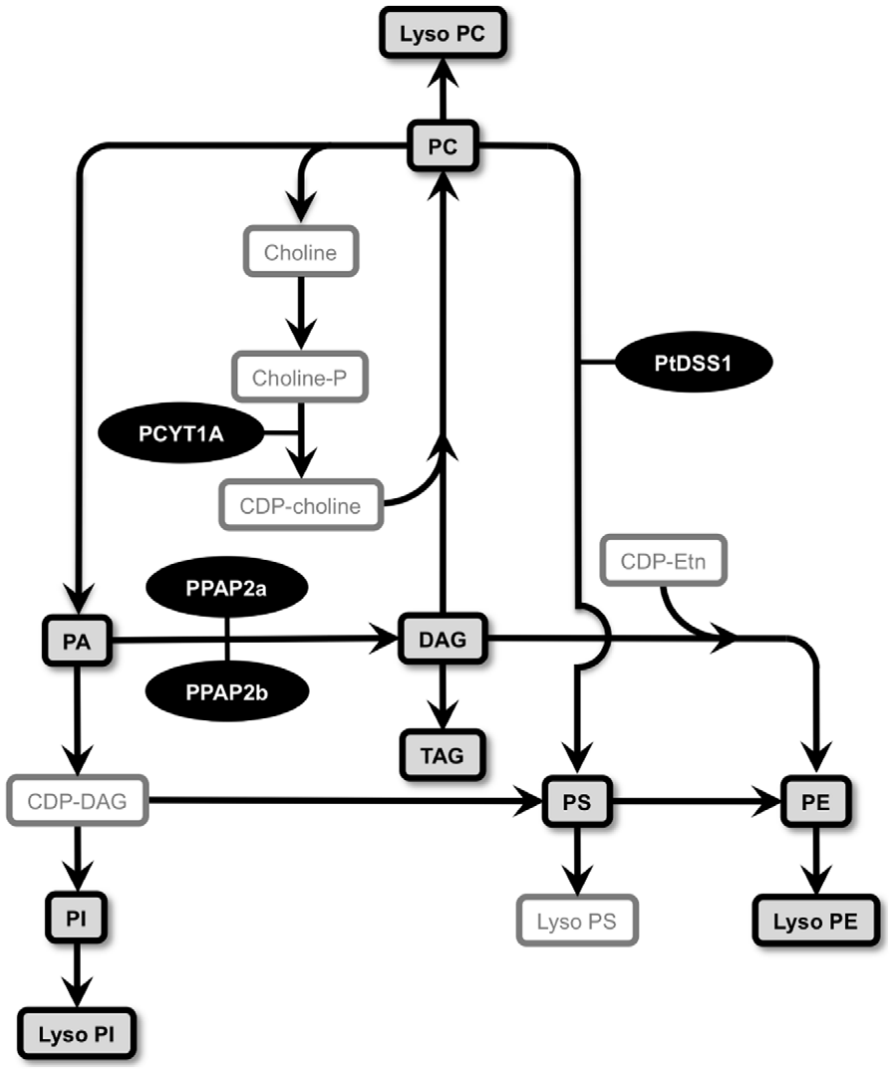
Simplified scheme of relevant glycerophospholipids and related genes assessed in this study. A simplified schematic diagram of relevant glycerophospholipids and related genes assessed in this study. The lipids in the boxes with black borders were analysed in the present study. Phosphatidylcholine (PC); phosphatidic acid (PA); phosphatidylinositol PI; phosphatidylserine PS; phosphatidylethanolamine (PE); diacylglycerol (DAG); cytidine diphosphate-diacylglycerol (CDP*-*DAG); cytidinediphosphate-choline (CDP*-*Choline); cytidinediphosphate-ethanolamine (CDP*-*Etn); phosphocholine cytidylytransferase 1a (PCYT1A); phosphatidic acid phosphatase 2a (PPAP2A); phosphatidic acid phosphatase 2B (PPAP2B); phosphatidylserine synthase I (PtDSS1).

**Figure 7:**
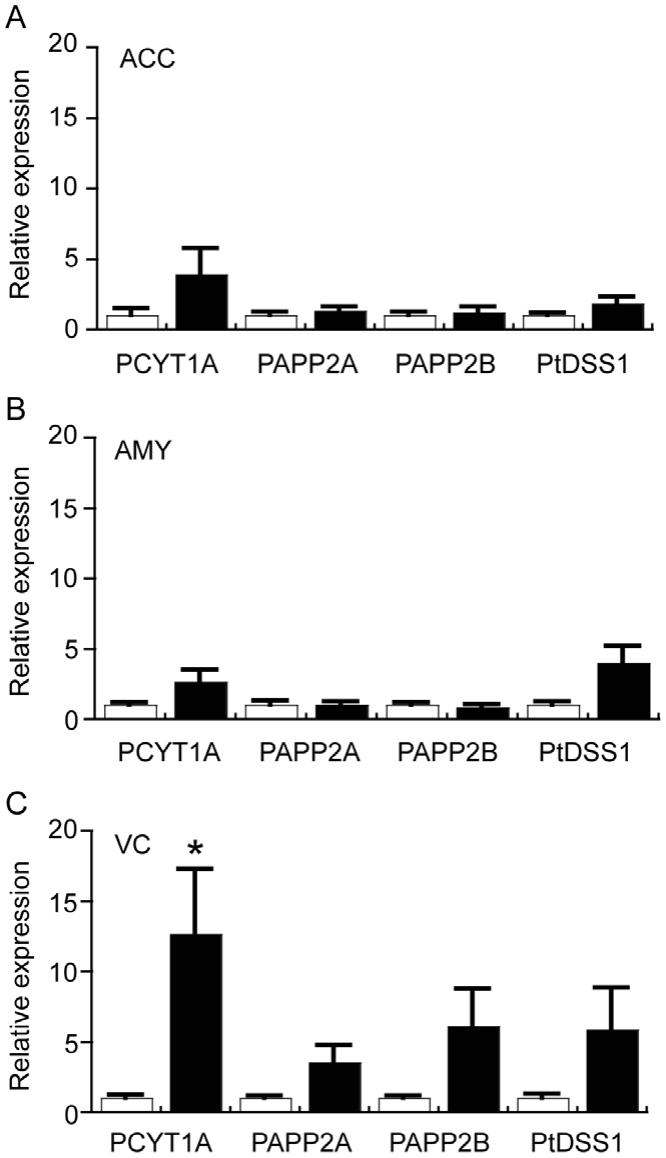
Quantitative real-time PCR analysis of selected glycerophospholipid-related genes in the anterior cingulate cortex (ACC), amygdala (AMY) and visual cortex (VC) of control (Con) and Parkinson's disease (PD) tissues. The expression of genes involved in the glycerophospholipid biosynthetic pathway (see Fig. 6) was assessed by qRT-PCR. Data for all genes are expressed relative to the control values (Con  =  white bars, assigned a value of 1.0; PD  =  black bars). The data are presented separately for ACC (A), AMY (B) and VC (C). Phosphocholine cytidylytransferase 1a (PCYT1A); Phosphatidic acid phosphatase 2a (PPAP2A); phosphatidic acid phosphatase 2B (PPAP2B); Phosphatidylserine synthase I (PtDSS1). Data represent mean ± SEM, *p<0.05 by *t*-test.

To understand the underlying mechanisms that may contribute to regional changes in glycerophospholipid metabolism in PD, we assessed the expression of a selection of relevant genes in the full sample cohort by qRT-PCR (as depicted in Figure 6). Of the genes investigated, the data indicated that PCYT1A was significantly up-regulated in the PD VC. This gene is important for the production of CDP*-*choline which is required to synthesise PC (Fig. 6). Interestingly, not all species of PC were increased in the PD VC and this may be due to conversion of specific molecular species of PC to PS. Consistent with this, PS levels were significantly increased in the PD VC and there was a non-significant trend for a 5-fold increase in the expression of the PTDSS1 gene that encodes for the enzyme required to catalyse this reaction (Fig. 7). Although DAG (classified here as a neutral lipid but also a crucial intermediate in glycerophospholipid synthesis, Fig. 6) levels were increased in the PD VC (Fig. 3), expression of two genes important for the synthesis of DAG from PA (PPAP2A and PPAP2B) were not significantly changed; although a trend for up-regulation was detected in the VC but not in the ACC or AMY (Fig. 7). Overall, the data point towards subtle modulation of the glycerophospholipid biosynthetic pathway to selectively modify glycerophospholipid profiles in the PD VC. The induction of the PCYT1A gene in the PD VC suggests that at least part of this change in lipid profile is transcriptionally regulated.

### Changes in neutral lipid and sterol metabolism related to PD

**Figure 8:**
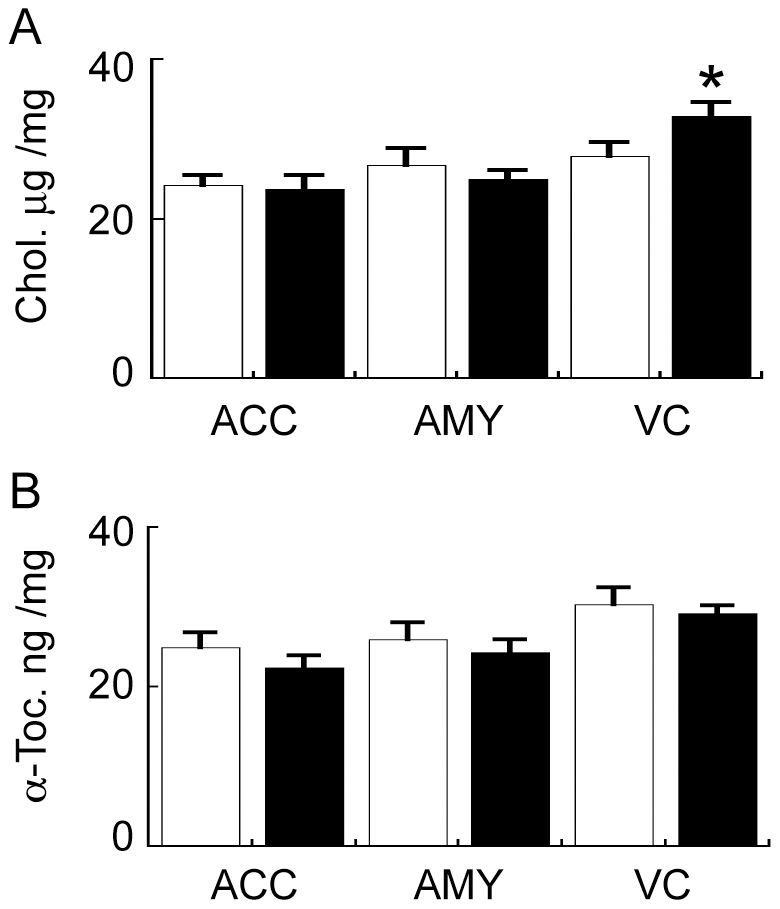
High performance liquid chromatography (HPLC) analysis of cholesterol and α-tocopherol in the anterior cingulate cortex (ACC), amygdala (AMY) and visual cortex (VC) of control (Con) and Parkinson's disease (PD) tissues. Cholesterol (A) and α-tocopherol (B) levels were analysed in the ACC, AMY and VC of Con (white bars) and PD (black bars) tissues by reversed phase HPLC. Data represent mean ± SEM, *p<0.05 by *t*-test.

The lipidomics LC/MS analysis indicated that TAG levels were decreased in the PD VC but not in the ACC or AMY as compared to the same brain regions derived from control samples (Fig. 3). This could also contribute to the increase in DAG detected in the PD VC (discussed above) as TAG are derived from DAG (Fig. 6). The lipidomics analysis also revealed an increase in cholesterol levels in the PD VC (Fig. 3). We re-assessed this by analysing an independent set of replicate samples by reversed phase HPLC. This analysis confirmed the lipidomics data (Fig. 8A), although the magnitude of increase in PD VC cholesterol was different (36% by LC/MS and 15% by HPLC). The lipid-soluble anti-oxidant α-tocopherol, which eluted from the HPLC column before cholesterol, was also quantified. α-Tocopherol levels were not significantly different in PD cases compared to controls in any of the brain regions examined (Fig. 8B). Age was not correlated with either cholesterol or α-tocopherol levels in any brain region (data not shown) and was therefore unlikely to confound the PD-related changes in cholesterol that we detected. The increase in PD VC cholesterol levels detected could theoretically be due to decreased formation of CE, however, only one of the ten CE molecular species analysed was found to be reduced (Fig. 3) and the relative amount of CE present in the brain is at least two orders of magnitude lower than cholesterol; so this is unlikely to be a major factor.

**Figure 9:**
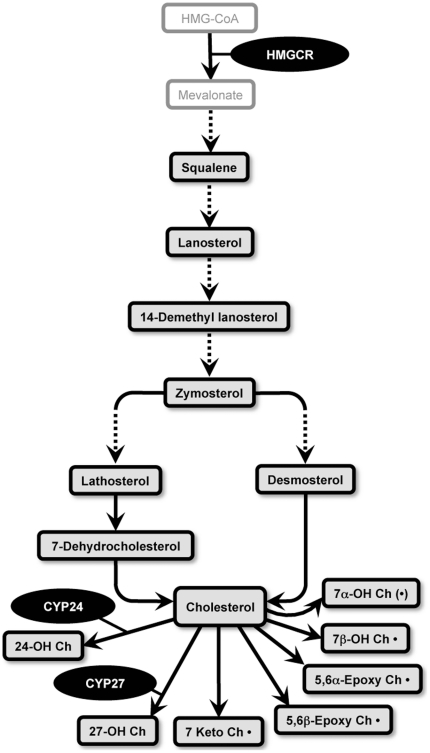
Simplified schematic diagram of cholesterol synthesis, cholesterol metabolites and selected relevant genes analysed in this study. The lipids in the boxes with black borders were analysed in the present study. The broken lines indicated additional intermediates are present in the pathway but they are not not shown in the scheme. The oxysterols that are followed by a dot “•” are formed by non-enzymatic oxidative reactions. The symbol “(•)” indicates the oxysterol is formed via both enzymatic and non-enzymatic routes. 24-hydroxycholesterol (24-OH Ch); 27-hydroxycholesterol (27-OH Ch); 7keto-cholesterol (7keto Ch); cholesterol-5α,6α-epoxide (5,6α-Epoxy Ch); cholesterol-5β,6β-epoxide (5,6β-Epoxy Ch); 7α-hydroxycholesterol (7α-OH Ch); 7β-hydroxycholesterol (7β-OH Ch); 3-hydroxy-3-methylglutaryl-CoA reductase (HMGCR); cytochrome P450, family 24, subfamily A, polypeptide 1 (CYP24); cytochrome P450, family 27, subfamily A, polypeptide 1 (CYP27).

**Figure 10:**
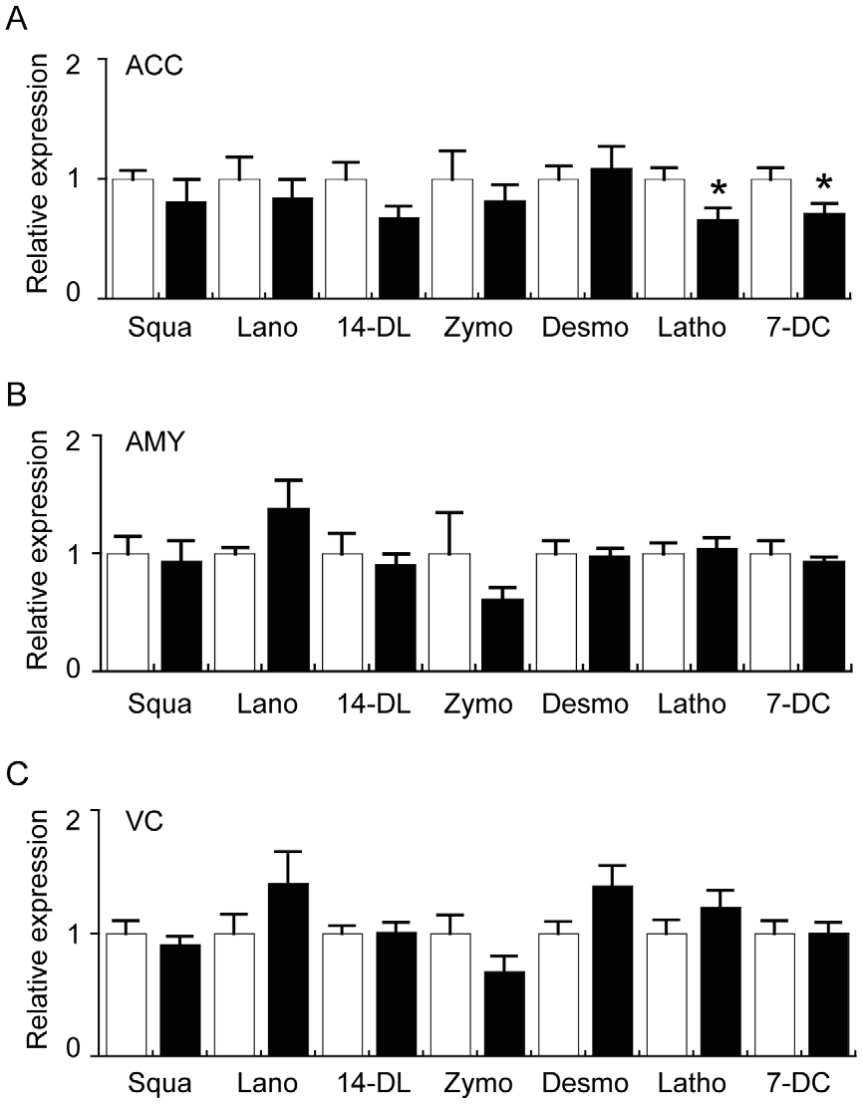
Gas chromatography mass spectrometry (GC/MS) analysis of cholesterol precursors in the anterior cingulate cortex (ACC), amygdala (AMY) and visual cortex (VC) of control (Con) and Parkinson's disease (PD) tissues. Control (n = 10) and PD (n = 10) tissues were collected from the ACC (A), AMY (B) and VC (C). Lipids were extracted and cholesterol precursors analysed using GC/MS. The data indicates the fold change in the levels of cholesterol precursors detected in the PD (black bars) samples relative to the Con (white bars) samples. Absolute values for the cholesterol precursors in the different brain regions are provided as Table S8. Squalene (Squa); lanosterol (Lano), 14-dimethyl lanosterol (14-DL), zymosterol (Zymo), desmosterol (Desmo), lathosterol (Latho), 7-dehyrocholesterol (7-DC). Data represent mean ± SEM.

In order to understand if cholesterol synthesis or metabolism may be altered in the PD VC, a further investigation of cholesterol biosynthetic precursor molecules as well as a range of oxysterol metabolites, that are indicated by the scheme depicted in Figure 9, was carried out using GC/MS analysis of the full sample cohort. There were no significant increases in any of the seven cholesterol precursor molecules assessed in any of the brain regions (Fig. 10). Intriguingly, lathosterol and 7-dehydrocholesterol levels were significantly reduced in the PD ACC compared to the control ACC (Fig. 10). The significance of this finding is not clear since the cholesterol levels were not different in the PD ACC versus control ACC (Figs. 3 and 8). Lathosterol was the only cholesterol precursor found to be correlated with age. This correlation was weak and only observed in the ACC (r^2^ = −0.23, *p* = 0.031). Age did not appear to have a major impact on the magnitude of PD-related differences in ACC lathosterol levels we detected in the full sample cohort as very similar data were generated using the age-matched samples (i.e. in the full cohort lathosterol levels were 24.1±2.4 ng/mg and 16.1±2.3 in the Con (n = 10) and PD (n = 10) groups, respectively (*t*-test *p* = 0.026); whereas in the age-matched cohort lathosterol levels were 23.6±2.8 ng/mg and 16.8±2.6 in the Con (n = 8) and PD (n = 8) groups, respectively (*t*-test *p* = 0.097).

One possible explanation for the lack of correlation between the levels of cholesterol and its precursors in the ACC and VC could be due to different rates of conversion of cholesterol to oxysterols in the different groups. To assess this, GC/MS was used to measure several oxysterols that are formed by free-radical mediated oxidation (7α-OH-Ch, 7β-OH-Ch, 5,6α-epoxy-Ch, 5,6β-epoxy-Ch, 7keto-Ch) or by enzymatic pathways (7α-OH-Ch, 27-OH-Ch, 24S-OH-Ch) [note: 7α-OH-Ch may be formed by both enzymatic and oxidative routes [60]].

**Figure 11:**
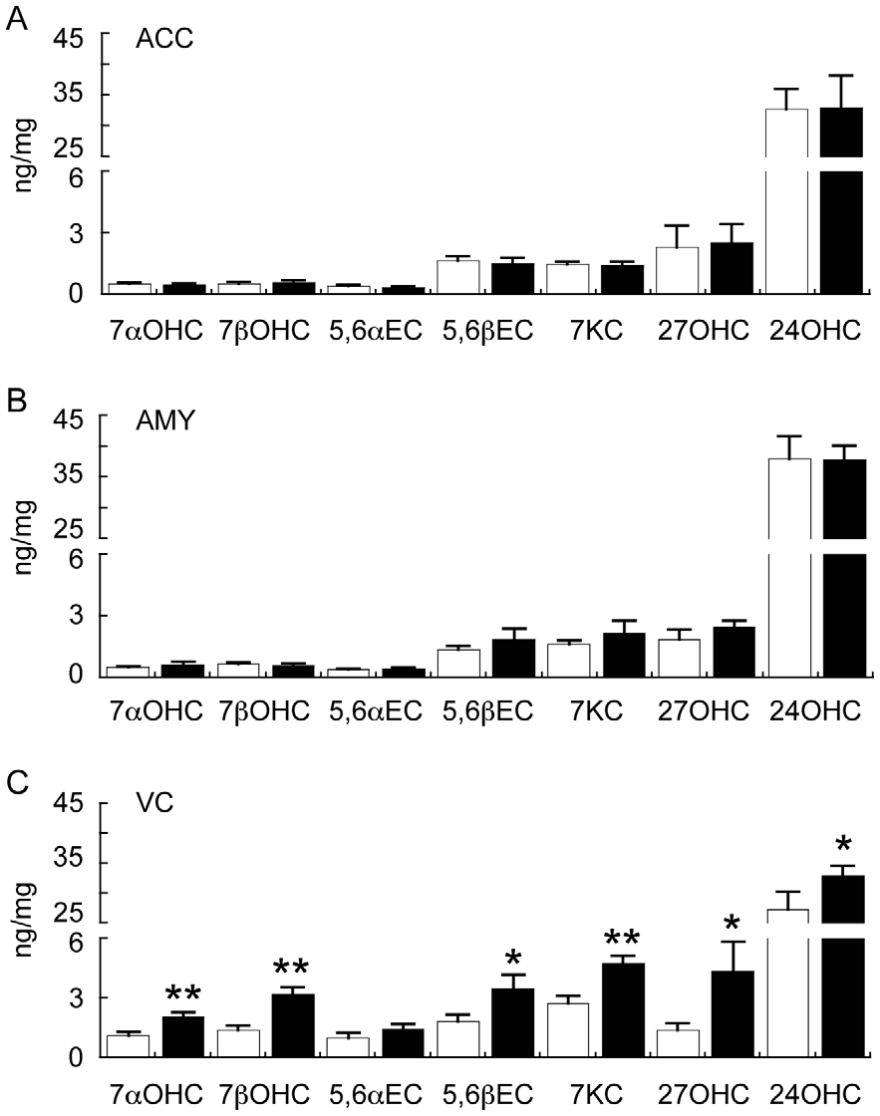
Gas chromatography mass spectrometry (GC/MS) analysis of oxysterols in the anterior cingulate cortex (ACC), amygdala (AMY) and visual cortex (VC) of control (Con) and Parkinson's disease (PD) tissues. Control (n = 10) and PD (n = 10) tissues were collected from the ACC (A), AMY (B) and VC (C). Lipids were extracted and oxysterols analysed using GC/MS. The data indicates the fold change in the levels of oxysterols detected in the PD (black bars) samples relative to the Con (white bars) samples. 7α-hydroxycholesterol (7αOHC); 7β-hydroxycholesterol (7βOHC); cholesterol-5α,6α-epoxide (5,6αEC); cholesterol-5β,6β-epoxide (5,6βEC); 7keto-cholesterol (7KC); 27-hydroxycholesterol (27OHC); 24-hydroxycholesterol (24OHC). Data represent mean ± SEM, *p<0.05, **p<0.001 by *t*-test.

The GC/MS analysis revealed a significant increase in PD VC oxysterols derived from both enzymatic and non-enzymatic routes (Fig. 11). Of the seven oxysterols analysed, 5,6α-epoxy-Ch was the only compound that was not significantly increased (although there was a trend for an increase) in the PD VC (Fig. 11). 7Keto-Ch was the only oxysterol found to be correlated with age. This correlation was weak and only observed in the VC (r^2^ = 0.20, *p* = 0.049). Similar to the observations regarding lathosterol above, age did not have an impact on the magnitude of PD-related differences in VC 7keto-Ch levels we detected in the full sample cohort as very similar data were generated using the age-matched samples (i.e. in the full cohort 7keto-Ch levels were 2.74±0.40 ng/mg and 4.73±0.40 in the Con (n = 10) and PD (n = 10) groups, respectively (*t*-test *p* = 0.002); whereas in the age-matched cohort 7keto-Ch levels were 2.83±0.49 ng/mg and 4.86±0.49 in the Con (n = 8) and PD (n = 8) groups, respectively (*t*-test *p* = 0.011).

Our finding that the levels of several oxysterols are increased in the PD VC raises two important issues. Firstly, it suggests that the small increase in total cholesterol levels detected in the PD VC (Fig. 8) is not due to decreased conversion to oxysterols such as 24S-OH-Ch; and secondly, the increase in non-enzymatic oxidation products implies the PD VC may be under a state of oxidative stress. To explore these issues, we conducted further analysis of gene expression in the full cohort.

**Figure 12:**
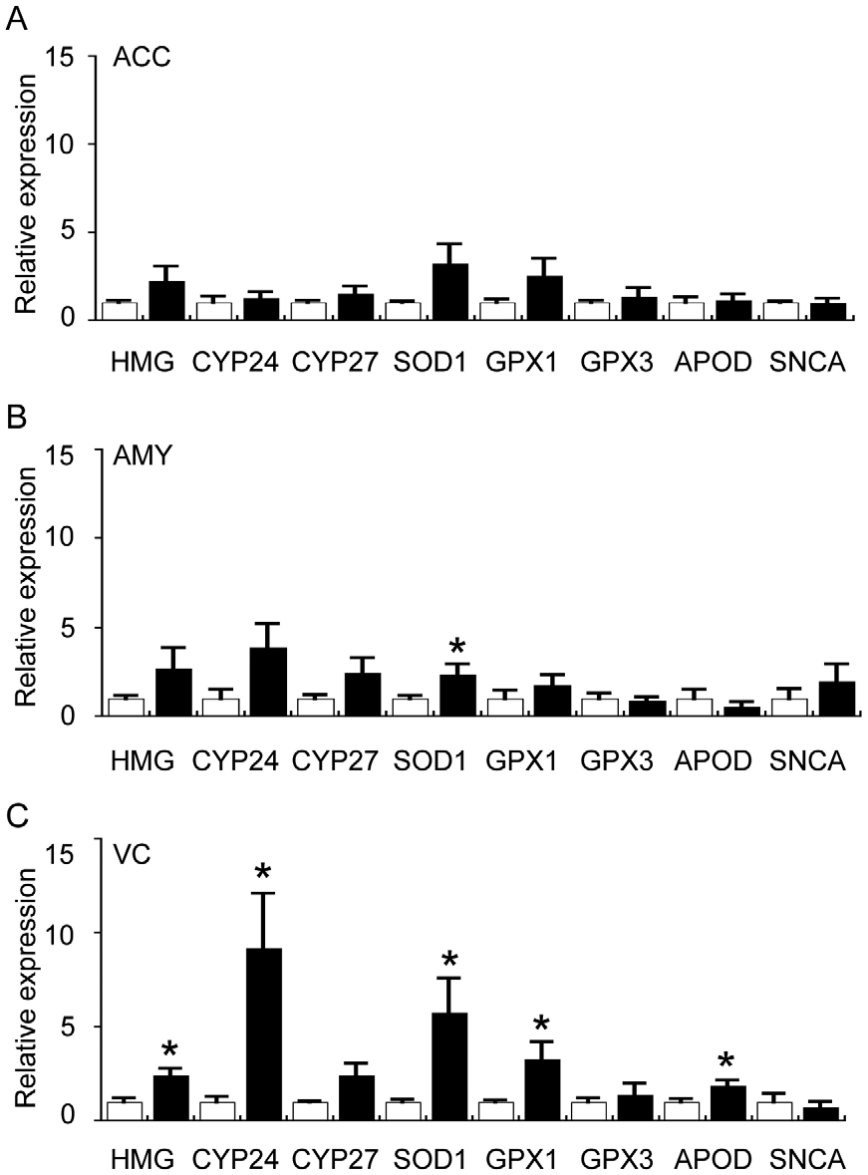
Quantitative real-time PCR analysis of selected sterol-related and oxidative stress-related genes in the anterior cingulate cortex (ACC), amygdala (AMY) and visual cortex (VC) of control (Con) and Parkinson's disease (PD) tissues. The expression of genes involved in cholesterol metabolism and oxidative stress was assessed by qRT-PCR. Data for all genes are expressed relative to the control values (Con  =  white bars, assigned a value of 1.0; PD  =  black bars). The data are presented separately for ACC (A), AMY (B) and VC (C). 3-hydroxy-3-methyl-glutaryl-CoA reductase (HMG); cytochrome P450, family 24, subfamily A, polypeptide 1 (CYP24); cytochrome P450, family 27, subfamily A, polypeptide 1 (CYP27); superoxide dismutase 1 (SOD1); glutathione peroxidase 1 (GPX1); glutathione peroxidase 3 (GPX3); Apolipoprotein-D (APOD); α-synuclein (SNCA). Data represent mean ± SEM, *p<0.05 by *t*-test.

We first examined the expression of HMGCR, the rate-limiting gene controlling cholesterol synthesis, and trends for increases were detected in all regions of the PD brain examined although this increase was significant only for the PD VC (Fig. 12). This is in general agreement with the lipidomics and HPLC data that indicated a statistically significant elevation of cholesterol only in the PD VC (Figs. 3 and 8). In addition, CYP24 expression was significantly increased in the PD VC (which may explain the increased levels of 24S-OH-Ch detected by GC/MS) whereas there was only a trend for increased CYP27 expression (Fig. 12). Interestingly, increases in SOD1, GPX1 and APOD gene expression were detected in the PD VC (Fig. 12). SOD1, APOD and specific GPX genes are also up-regulated in the human brain under oxidative stress conditions and have been shown to be correlated with increases in markers of brain lipid peroxidation in the human prefrontal cortex during development and ageing and in the SN in PD [55], [61].

We also used GC/MS to analyse levels of F2-isoprostanes (sensitive markers of arachidonic acid oxidation) and found no evidence for an increase in any of the PD samples (A. Jenner, D. Cheng and B. Garner, unpublished observations). This, along with the fact that α-tocopherol levels were not depleted in the PD tissues (Fig. 8), implies that the increase in oxidative stress in the PD VC is not a “generalised” condition but may be rather specific (e.g. perhaps limited to cholesterol). As it has been shown previously that 27-OH-Ch up-regulates neuronal α-syn mRNA expression [62], we also assessed SYNA gene expression and the results indicate there were no differences related to either 27-OH-Ch levels or to PD status (Fig. 12). The failure of the increased levels of 27-OH-Ch present in the PD VC to induce α-syn transcription may be due to a complex interplay with 24S-OH-Ch as another study has suggested that 27-OH-Ch-mediated up-regulation of SYNA can be blocked in the presence of equimolar 24S-OH-Ch [63].

## Discussion

The data presented herein represent the first lipidomics analysis of the human PD brain. Although previous work focused on lipid changes detectable in the SN using histochemical techniques [5], we have chosen not to focus our present study on the SN for two reasons. Firstly, the extensive neuronal loss and gliosis that occurs in the SN in association with PD would obscure interpretation of any changes in lipid metabolism that may be revealed by a lipidomics analysis [3], [7], [64], and secondly, the loss of neuromelanin from the SN in PD will similarly confound the lipid analysis as this pigment is enriched in lipids including cholesterol and, in particular, dolichol [54].

The most important findings arising from our current work relate to changes in lipid metabolism in the PD VC. It should be emphasised that there are no substantive pathological changes observed in this region of the brain in PD; although previous studies have indicated changes in metabolic activity by PET and fMRI techniques [20]–[23]. Our data indicate an activation of the sphingolipid biosynthetic pathway in the PD VC that appears to be regulated at the transcriptional level. Increases in specific ceramide species and other sphingolipids may alter intracellular signalling and contribute to neuronal dysfunction in PD [65]. Similarly, alterations in DAG and glycerophospholipid metabolism may also modulate neuronal function in the PD VC [66]. We speculate that such significant changes in lipid metabolism might result in neuronal dysfunction of the primary VC in PD and that these changes may impact on visual perception and possibly contribute to VH.

Oxidative stress resulting in lipid peroxidation has been suggested to cause neuronal death in the PD SN [67], [68]. Cholesterol is highly enriched in the brain and previous studies have shown that specific oxysterols (e.g. 7β-OH-Ch and 24S-OH-Ch) may be neurotoxic at reasonably high µM concentrations [69], [70]. At sub-lethal concentrations, oxysterols including 27-OH-Ch and 24S-OH-Ch can also regulate the transcription of α-syn and many other genes involved in neuroinflammation and neurodegeneration via the liver-X-receptor (LXR) pathway [62], [63], [71]–[73]. Very recently, oxysterol-mediated LXR activation of human embryonic stem cells (hESC) has been shown to increase neurogenesis and this led to the suggestion that oxysterols may be used to improve hESC replacement strategies for PD [74]. Therefore, although the formation of oxysterols could be considered deleterious, it is possible that increases in certain oxysterols we have observed in the VC may perform a function that is protective in the PD context.

Another consideration is the source of oxysterol increase we have detected in the PD VC. Our assumption is that the oxysterols are mostly locally produced, and this would fit with the changes in CYP24 expression that our data indicate are correlated with increased 24S-OH-Ch; however, it is quite likely that the increase in 27-OH-Ch we detected could also be due to plasma derived oxysterol. Relevant to this point, recent studies have shown that 27-OH-Ch levels are significantly elevated in PD plasma [75], [76]. Similar ideas have been put forward regarding the source of elevated 27-OH-Ch levels in Alzheimer's disease brain where, intriguingly, levels of this oxysterol are also elevated in the occipital cortex [77], [78].

Our current study has also revealed for the first time that there is an up-regulation of antioxidant response genes in the PD VC. Oxidative stress has been well established as a causative factor in the pathways that result in neuron loss within the SN in PD [67]; however, due to the lack of pathology in the VC, the up-regulation of genes such as SOD1, GPX1 and APOD that we detected was rather unexpected. It is possible that hypoperfusion of the PD VC that is associated with hypometabolism of glucose could result in less efficient production of reducing equivalents through the Krebs cycle and thereby place the origin of the oxidative stress response at the level of metabolic changes in the mitochondria (of neurons and/or astroglial cells) [79], [80]. Interestingly, previous work has shown that glial apoD and GPX1 levels are increased in the SN in PD cases [61], [81], [82]. From these studies it was concluded that glia may afford neuroprotection in the SN and it remains possible that a similar process occurs in the VC in PD.

It is currently not known if the changes we have detected in lipid homeostasis and oxidative stress in the VC are specific for PD. As noted above, oxysterol metabolism is altered in the occipital cortex in Alzheimer's disease [77], [78]. There is also evidence that brain injury induced by trauma or stroke alters lipid peroxidation status [83]–[85]. Furthermore, data from animal studies indicate that bioactive oxysterols, such as 24S-OH-Ch, increase in response to traumatic brain injury and this can modulate the transcription of specific genes that regulate lipid homeostasis [86]. Interestingly, it has been reported that visual hallucinations are present in ∼30% of Alzheimer's disease patients [87]. Similarly, VH have been reported in association with both stroke and traumatic brain injury [88], [89]. Based on the fact that alterations in cerebral lipid homeostasis and oxidative stress status appear to coexist with VH in other neurological conditions, we cannot exclude the possibility that the changes in lipid homeostasis and oxidative stress we have detected in the PD VC may be a generalised phenomenon that is not specific to PD VH. Further studies that compare cerebral lipid homeostasis in large cohorts of PD patients both with and without VH would provide further evidence for a specific association.

In conclusion, our studies reveal significant alterations in the sphingolipid and glycerophospholipid pathways in the primary VC in idiopathic PD. We also show that cholesterol metabolism through the oxysterol pathway is up-regulated in the PD VC. These changes are associated with selective changes in the expression of genes responsible for the control of lipid biosynthesis and with increased expression of antioxidant genes. Although causation can not be established from these studies, the data do suggest that normalization of the dysregulated lipid metabolism/oxidative stress status in the VC may represent a novel route for treatment of the VH that are experienced by a majority of PD patients.

## Supporting Information

Figure S1 **Workflow of lipidomic screening strategy.** The strategy for human brain tissue sample collection, distribution and analysis of the major lipid classes is illustrated.
(TIF)

Figure S2 **Analysis of α-synuclein is SDS fraction Parkinson's disease amygdala.** Tissues were homogenised into three fractions that contained tris-buffered saline, TBS containing Triton X100 or sodium dodecyl sulphate (SDS) and α-synuclein (α-Syn) expression was analysed by Western blotting. The detection of high molecular weight species of α-syn are indicated, the arrow indicates a 31 kDa α-Syn band that is present in some of the human brain SDS fractions. The data are derived from Parkinson's disease amygdala (PD AMY) samples.
(TIF)

Table S1 **qRT-PCR primer details.** Primers were designed using Primer3 software (available at http://www.ncbi.nlm.nih.gov/) and based on the National Center for Biotechnology Information (NCBI) reference sequences. The specificity of the primers was confirmed by demonstration of a single PCR product of the correct size as judged by agarose gel electrophoresis.
(DOC)

Table S2 **Changes in lipid levels associated with Parkinson's disease (PD) anterior cingulate cortex as assessed using a lipidomics approach.** Control (n = 10) and PD Patient (n = 10) tissues were collected from the anterior cingulate cortex (ACC). Lipids were extracted and analysed using an LC/MS lipidomics approach. The data indicates mol% values for all lipid species listed in the Table. The fold-change values in the levels of sphingolipids, glycerophospholipids and neutral lipids detected in the PD patient samples relative to the Con samples are provided (“Ratio”) along with the *t*-test *p*-values. These data were used to generate the heat map provided in Figure 3.
(XLS)

Table S3 **Changes in lipid levels associated with Parkinson's disease (PD) amygdala as assessed using a lipidomics approach.** Control (n = 10) and PD Patient (n = 10) tissues were collected from the amygdala (AMY). Lipids were extracted and analysed using an LC/MS lipidomics approach. The data indicates mol% values for all lipid species listed in the Table. The fold-change values in the levels of sphingolipids, glycerophospholipids and neutral lipids detected in the PD patient samples relative to the Con samples are provided (“Ratio”) along with the *t*-test *p*-values. These data were used to generate the heat map provided in Figure 3.
(XLS)

Table S4 **Changes in lipid levels associated with Parkinson's disease (PD) primary visual cortex as assessed using a lipidomics approach.** Control (n = 10) and PD Patient (n = 10) tissues were collected from the primary visual cortex (VC). Lipids were extracted and analysed using an LC/MS lipidomics approach. The data indicates mol% values for all lipid species listed in the Table. The fold-change values in the levels of sphingolipids, glycerophospholipids and neutral lipids detected in the PD patient samples relative to the Con samples are provided (“Ratio”) along with the *t*-test *p*-values. These data were used to generate the heat map provided in Figure 3.
(XLS)

Table S5 **False discovery rate (FDR) analysis for the lipidomics data sets.** False discovery rate (FDR) *q*-values were calculated from the *t*-test *p-*values for the lipidomics datasets provided in Tables S2 to S4. Data are sorted based on *p*-value ascending order. VC, visual cortex; AMY, amygdala; ACC, anterior cingulate cortex.
(XLS)

Table S6 **Comparison of electrospray ionisation mass spectrometry and liquid chromatography mass spectrometry data.** All values represent the fold-change in PD lipids relative to the control cases. UOW ESI/MS, University of Wollongong electrospray ionisation mass spectrometry; National University of Singapore, liquid chromatography mass spectrometry.
(DOC)

Table S7 **Brain sphingolipid quantification by electrospray ionisation mass spectrometry.** Semi-quantitative analysis of the major sphingolipid species present in Control and PD brain samples was assessed by ESI/MS. All values are nmol/g tissue (wet weight). Note that changes in pulverized tissue sample moisture content during storage of samples at −80°C may influence the absolute quantities of lipids given in the Table.
(DOC)

Table S8 **Cholesterol precursor quantification by gas chromatography mass spectrometry.** Semi-quantitative analysis of the major cholesterol precursor species present in brain samples was assessed by GC/MS. All values are ng/mg tissue (wet weight). Note that changes in pulverized tissue sample moisture content during storage of samples at −80°C may influence the absolute quantities of lipids given in the Table.
(XLS)

Methods S1 **Detailed methods.**
(DOC)
