# Supplementary figures and images for: Lipid Pathway Alterations in Parkinson's Disease Primary Visual Cortex
Source: PLoS One. 2011 Feb 28;6(2):e17299. doi: 10.1371/journal.pone.0017299 (PMC3046155; doi:10.1371/journal.pone.0017299)

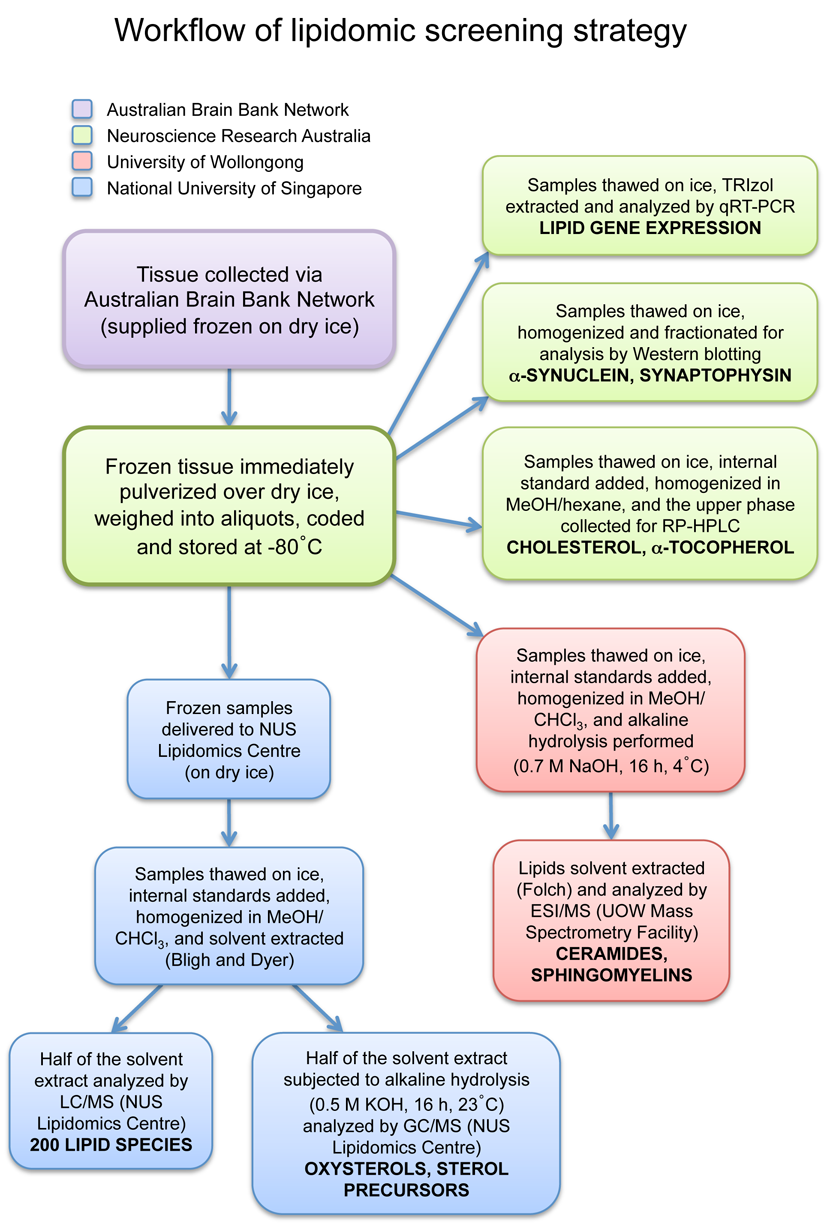

Supplement: Figure S1 — Workflow of lipidomic screening strategy. The strategy for human brain tissue sample collection, distribution and analysis of the major lipid classes is illustrated. (TIF) [file pone.0017299.s001.tif]

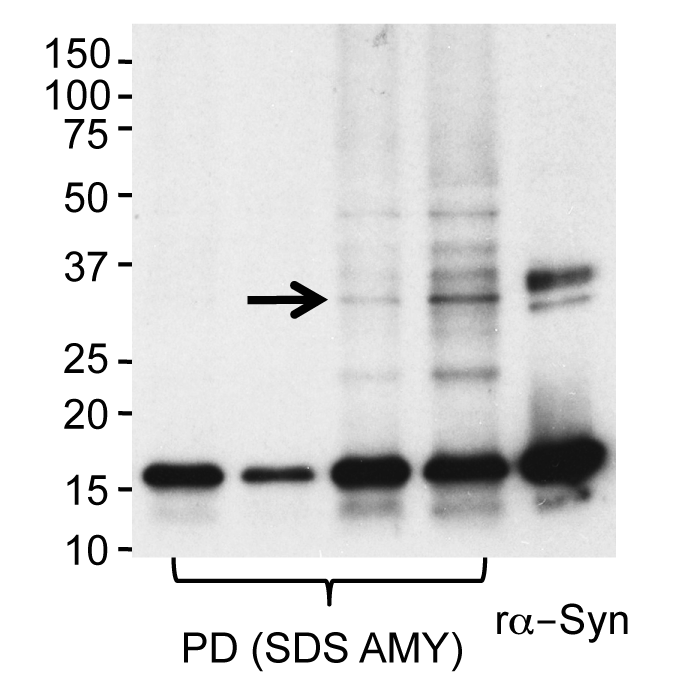

Supplement: Figure S2 — Analysis of α-synuclein is SDS fraction Parkinson's disease amygdala. Tissues were homogenised into three fractions that contained tris-buffered saline, TBS containing Triton X100 or sodium dodecyl sulphate (SDS) and α-synuclein (α-Syn) expression was analysed by Western blotting. The detection of high molecular weight species of α-syn are indicated, the arrow indicates a 31 kDa α-Syn band that is present in some of the human brain SDS fractions. The data are derived from Parkinson's disease amygdala (PD AMY) samples. (TIF) [file pone.0017299.s002.tif]
