# Supplementary material for: Lipid Pathway Alterations in Parkinson's Disease Primary Visual Cortex
Source: PLoS One. 2011 Feb 28;6(2):e17299. doi: 10.1371/journal.pone.0017299 (PMC3046155; doi:10.1371/journal.pone.0017299)
Supplement: Table S1 — qRT-PCR primer details. Primers were designed using Primer3 software (available at http://www.ncbi.nlm.nih.gov/) and based on the National Center for Biotechnology Information (NCBI) reference sequences. The specificity of the primers was confirmed by demonstration of a single PCR product of the correct size as judged by agarose gel electrophoresis. (DOC) [file pone.0017299.s003.doc]

| Primer | Forward | Reverse |
| --- | --- | --- |
| PCYT1B | TCACCGTGATGAATGAAGCCGAG | TCTGGCGTGAGTGTCCAGGGA |
| PAPP2A | GGCCCTCGATGTGCTCTGCG | CCTCGTTGGAAGGGGGTATGCC |
| PAPP2B | GCAGCCAGCGCCATGCAAAA | GGCCCGCCATGAAGAGGCAG |
| PTDSS1 | GACGGGGAGGCGGGCCATG | GGTGATGGTATGCGGCCGGT |
| SPTLC2 | GGCCGTGAAGAAAGAATGTGAGTAACTG | CAAGAGAGGAAAGATACCTTGGGAATTAGG |
| FVT1 | GAGCGATGCTGCTGCTGGCT | GGCTTGGGGCTGATGAGCGG |
| DEGS1 | GGGTCTACACCGACCAGCCG | CTGCCAAACGCATAGGCCCC |
| SGMS1 | TCGGAACAGTGACTGCTGAC | GAAATGCTCCAGAGGCTCAC |
| UGT8A | GGGGCAGCCAAGAGACGAGC | TGGGTAGCGCTGGAGGCTGT |
| GAL3ST1 | AGACGCACAAGACGGCCAGC | GATGGCGTTGGTCGGCACCA |
| HMGCoAR | CAAGGAGCATGCAAAGATAATCC | GCCATTACGGTCCCACACA |
| CYP24A | TGTGCATTGGTCGCCGATTAGC | TCGCTGGCAAAACGCGATGG |
| CYP27A | CGGCGGCAACGGAGCTTAGA | GCTCCAAGAGCGGGGCACTG |
| SOD1 | GGCAAAGGTGGAAATGAAG | GCAACTCTGAAAAAGTCACAC |
| GPX1 | ACCACGGTCCGGGACTAC | CCGGACGTACTTGAGGGAAT |
| GPX3 | GCCGGGGACAAGAGAAGT | GAGGACGTATTTGCCAGCAT |
| ApoD | ctgcatccaggccaactact | tggatgatgcaggtacagga |
| αSyn | TAGGCTCCAAAACCAAGGAGG | CCTTCTTCATTCTTGCCCAACT |
| β-actin | TCATGAAGTGTGACGTGGACATCCGT | CCTAGAAGCATTTGCGGTGCACGATG |

**Supplemental Table 1. qRT-PCR primers**
