# Supplementary material for: Lipid Pathway Alterations in Parkinson's Disease Primary Visual Cortex
Source: PLoS One. 2011 Feb 28;6(2):e17299. doi: 10.1371/journal.pone.0017299 (PMC3046155; doi:10.1371/journal.pone.0017299)
Supplement: Table S6 — Comparison of electrospray ionisation mass spectrometry and liquid chromatography mass spectrometry data. All values represent the fold-change in PD lipids relative to the control cases. UOW ESI/MS, University of Wollongong electrospray ionisation mass spectrometry; National University of Singapore, liquid chromatography mass spectrometry. (DOC) [file pone.0017299.s008.doc]

**Supporting Information Table S6. Comparison of ESI/MS and LC/MS data**

| ***, p <0.05**  **t-test** | UOW ESI/MS | | | NUS LC/MS | | |
| --- | --- | --- | --- | --- | --- | --- |
| Lipids | ACC | AMY | VIS | ACC | AMY | VIS |
| SM18:1/16:0 | 0.86 | 1.19 | 1.62 | 1.34 | 0.96 | 1.08 |
| SM18:1/18:1 | 0.84 | 1.06 | 1.26 | 0.95 | 0.98 | 1.08 |
| SM 18:1/18:0 | 1.01 | 1.10 | ***1.36** | 0.97 | 1.00 | 1.00 |
| SM 18:1/20:0 | 0.86 | 0.92 | ***0.83** | 0.96 | 0.91 | 0.85 |
| SM18:1/22:0 | 1.00 | 1.36 | ***2.08** | 1.24 | 1.03 | ***1.25** |
| SM18:1/24:1 | 1.04 | 1.12 | ***2.32** | 0.95 | 1.09 | ***1.36** |
| Cer d18:0/18:0 | 1.20 | ***1.47** | ***1.48** | 2.16 | ***1.59** | ***2.07** |
| Cer d18:0/24:1 | 1.56 | 2.27 | ***3.88** | 1.21 | 1.72 | ***3.25** |

All values represent the fold change in PD lipids relative to the control cases.
