# Supplementary material for: Lipid Pathway Alterations in Parkinson's Disease Primary Visual Cortex
Source: PLoS One. 2011 Feb 28;6(2):e17299. doi: 10.1371/journal.pone.0017299 (PMC3046155; doi:10.1371/journal.pone.0017299)
Supplement: Table S7 — Brain sphingolipid quantification by electrospray ionisation mass spectrometry. Semi-quantitative analysis of the major sphingolipid species present in Control and PD brain samples was assessed by ESI/MS. All values are nmol/g tissue (wet weight). Note that changes in pulverized tissue sample moisture content during storage of samples at −80°C may influence the absolute quantities of lipids given in the Table. (DOC) [file pone.0017299.s009.doc]

Supporting data Table S7. Semi-quantitative analysis of major sphingolipid species by ESI/MS

|  | Anterior Cingulate Cortex (nmol/g) | | | |
| --- | --- | --- | --- | --- |
|  | Con (n=10) | | PD (n=10) | |
|  | Mean | SE | Mean | SE |
| Cer d18:0/18:0 | 58.02 | 4.36 | 69.57 | 4.91 |
| Cer d18:0/24:1 | 24.71 | 7.78 | 28.10 | 11.95 |
| SM 18:1/16:0 | 122.21 | 14.57 | 104.48 | 12.62 |
| SM 18:1/18:1 | 170.03 | 14.24 | 142.04 | 10.80 |
| SM 18:1/18:0 | 1114.67 | 80.60 | 1125.67 | 60.10 |
| SM 18:1/20:0 | 271.82 | 25.33 | 232.43 | 22.33 |
| SM 18:1/22:0 | 35.49 | 9.51 | 35.43 | 8.67 |
| SM 18:1/24:1 | 423.18 | 136.13 | 440.52 | 170.85 |
| SM 18:1/26:0 | 97.00 | 41.04 | 93.56 | 36.25 |

|  | Amygdala (nmol/g) | | | |
| --- | --- | --- | --- | --- |
|  | Con (n=10) | | PD (n=10) | |
|  | Mean | SE | Mean | SE |
| Cer d18:0/18:0 | 65.68 | 2.85 | 96.20 | 10.33 |
| Cer d18:0/24:1 | 15.03 | 3.08 | 34.12 | 10.00 |
| SM 18:1/16:0 | 169.71 | 16.32 | 201.71 | 24.14 |
| SM 18:1/18:1 | 214.38 | 12.58 | 216.58 | 16.91 |
| SM 18:1/18:0 | 1345.26 | 54.53 | 1486.93 | 84.82 |
| SM 18:1/20:0 | 234.54 | 13.40 | 214.64 | 12.23 |
| SM 18:1/22:0 | 36.56 | 3.99 | 49.87 | 11.05 |
| SM 18:1/24:1 | 576.63 | 81.71 | 645.53 | 143.33 |
| SM 18:1/26:0 | 104.45 | 7.00 | 173.96 | 43.15 |

|  | Visual Cortex (nmol/g) | | | |
| --- | --- | --- | --- | --- |
|  | Con (n=10) | | PD (n=10) | |
|  | Mean | SE | Mean | SE |
| Cer d18:0/18:0 | 65.90 | 5.44 | 97.60 | 9.41 |
| Cer d18:0/24:1 | 17.42 | 4.14 | 67.51 | 21.25 |
| SM 18:1/16:0 | 94.44 | 4.38 | 155.32 | 29.89 |
| SM 18:1/18:1 | 113.56 | 6.71 | 120.85 | 15.78 |
| SM 18:1/18:0 | 914.59 | 45.34 | 1248.38 | 150.27 |
| SM 18:1/20:0 | 263.39 | 18.88 | 218.89 | 11.05 |
| SM 18:1/22:0 | 30.52 | 1.88 | 63.63 | 13.74 |
| SM 18:1/24:1 | 500.52 | 50.47 | 1162.04 | 295.21 |
| SM 18:1/26:0 | 76.50 | 7.00 | 183.95 | 43.15 |
