# Supplementary material for: Lipid Pathway Alterations in Parkinson's Disease Primary Visual Cortex
Source: PLoS One. 2011 Feb 28;6(2):e17299. doi: 10.1371/journal.pone.0017299 (PMC3046155; doi:10.1371/journal.pone.0017299)
Supplement: Methods S1 — Detailed methods. (DOC) [file pone.0017299.s011.doc]

Supporting Information, Cheng at al.

Detailed methods

**Brain sample collection details**

Following brain only autopsy, the brains were transported to the same research laboratory for standardised processing that was photographically recorded. Each brain was separated midsagitally and one half sectioned into 0.5 - 1 cm coronal slices. Standardised blocks were taken for flash freezing and included the amygdala (AMY, in the anterior temporal lobe), the anterior cingulate cortex (ACC, the gyrus immediately posterior and superior to the genu of the corpus callosum), and the primary visual cortex (VC, the cortex surrounding the calcarine sulcus and containing a visible line of Genarri). All sampled blocks and the remaining coronally sectioned tissue were stored in -80ºC freezers until required. The other hemisphere was fixed for two weeks in formalin for histological case characterisation.

**Western blotting for α-syn and synaptophysin**

The brain tissue was homogenized into three fractions as described previously [1,2]. In brief, tissues were homogenized with a pre-chilled 2 mL glass dounce homogenizer, using 150 μL of ice-cold tris-buffered saline (TBS, pH 7.4) lysis buffer (LB) containing protease inhibitors (Roche, cat. # 1836153001, Dee Why, Australia) and phosphatase inhibitors (Thermo Fisher Scientific, cat. # PIE78420, Scoresby, Australia). After centrifugation at 16,000 *g* for 25 min at 4°C the TBS-soluble supernatant fraction was collected. The pellet was washed with 50 μL of TBS and centrifuged for another 5 min, the supernatant was discarded and the pellet resuspended in 150 μL LB containing 1% (w/v) Triton X-100 (TX). Samples were then mixed by rotation for 30 min at 4°C, followed by centrifugation at 16,000 *g* for 25 min at 4°C. The TX-soluble fraction was collected and the pellet centrifuged for another 5 min for removal of residual TX. Pellets were subsequently dissolved by vortex mixing in 400 μL LB containing 2% (w/v) SDS and collected as the SDS-soluble fraction.

Protein concentration of the brain homogenates was determined using the bicinchoninic acid (BCA) assay [1] and equal amounts of protein (routinely 20μg) were resolved on 12% SDS-PAGE gels and transferred onto 0.45 μm nitrocellulose membranes at 100 V for 30 min. Membranes were briefly stained with Ponceau Red, to confirm equal loading and transfer, before blocking in phosphate-buffered saline (PBS) containing 5% (w/v) non-fat dry milk. Subsequently, the membranes were probed with either α-syn antibody 1:1000 (mouse monoclonal, BD biosciences, North Ryde, Australia, cat. # 610787) or synaptophysin antibody 1:50000 (mouse monoclonal, Millipore, North Ryde, Australia, cat. # MAB368) for 2 h at 22°C. The membranes were washed three times in 0.1% Tween-20 PBS and then incubated with horseradish peroxidase (HRP) conjugated rabbit anti-mouse polyclonal antibody (1:2000, Dako, Carpinteria, CA, cat. # P0260) for 2 h at 22°C. Membranes were washed as previously stated and enhanced chemiluminescence detected using ECL Hyperfilm (GE Healthcare Biosciences, Buckinhamshire, England). To verify equal loading, blots were stripped in Restore Stripping Buffer (Quantum scientific, Lane Cove, Australia) and immunostained for β-actin (rabbit polyclonal, Sigma-Aldrich, Sydney, Australia, cat. # A5060) using goat anti-rabbit HRP-conjugated secondary antibody (1:2000, Dako, cat. # P0448). Signal intensities were quantified using NIH Image J software (National Institutes of Health, Bethesda, MD) with the relative expression of bands of interest normalised to β-actin.

**Analysis of lipids using high performance liquid chromatography/mass spectrometry (LC/MS)**

Lipids were extracted from tissue samples using a modified Bligh and Dyer double chloroform extraction method [3]. Brain tissues (~30mg) were homogenized in 0.9 mL ice-old organic solvent mixture (CHCl_3_/methanol, 1/2 v/v) containing internal standards and heavy isotopes (40 ng 7 α-hydroxycholesterol-d_7_, 40 ng 7 β-hydroxycholesterol-d_7_, 40 ng 14-demethyl-lanosterol-d_6_, 40 ng zymosterol-d_5_, 80 ng of 7-ketocholesterol-d_7_, 0.2 µg 5-α cholestane, 0.2 µg lathosterol-d_4_, 0.2 ug desmosterol-d_6_, 0.2 ug of 27-hydroxycholesterol-d_5_, 0.2 µg of campesterol-d_7_, and 0.2 µg of β-sitosterol-d_7_) and incubated on ice with shaking for 2 h in a vacuum container in a 4˚C dark room. 0.3 mL of chloroform and 0.45 mL of ice-cold water were added into the homogenate. The mixture was vortexed for 30 sec and centrifuged at 16,000 *g* for 5 min, the lower organic layer was carefully transferred to an Eppendorf tube. 0.5 ml of ice-cold chloroform was added for a second extraction. The two extracts were pooled, split into two aliquots for LC/MS and GC/MS (see below) analysis, dried under a stream of N_2_ and stored at -80°C until lipid analysis.

An Agilent high performance liquid chromatography (HPLC) 1200 system coupled with an Applied Biosystem Triple Quadrupole/Ion Trap mass spectrometer (3200 Qtrap) was used for quantification of individual phospholipids and sphingolipids [4,5]. Separation of individual lipid classes of polar lipids was carried out using a Luna 3 μm silica column (i.d. 150 x 2.0 mm). HPLC conditions were: mobile phase A (chloroform: methanol: ammonium hydroxide, 89.5:10:0.5), B (chloroform: methanol: ammonium hydroxide:water, 55:39:0.5:5.5); flow rate 300 µl/min; 5% B for 3 min, then linearly changed to 30% B in 24 min and maintained for 5 min, and then linearly changed to 70% B in 5 min and maintained for 7 min. Then, the eluents were changed to the original ratio in 5 min and maintained for 6 min. Multiple reaction monitoring (MRM) transitions were set up for quantitative analysis of various polar lipids [5-7]. Levels of individual lipids were quantified using spiked internal standards. PC-14:0/14:0, PE-14:0-14:0, PS-14:0/14:0, PA-17:0/17:0, PG-14:0/14:0, d31-PI-18:1/16:0, C17-sphingosine, C17-Cer, C12-sulfatide and C12-SM were obtained from Avanti Polar Lipids (Alabaster, AL, USA). Dioctanoyl phosphatidylinositol (PI, 16:0-PI) was used for phosphatidylinositol quantitation and obtained from Echelon Biosciences, Inc. (Salt Lake City, UT, USA).

Neutral lipids were analyzed using a modified method from a previously described LC/MS method [6]. Briefly, separation of triglycerides (TGs) from polar lipids was carried out on an Agilent Zorbax Eclipse XDB-C18 column (i.d. 4.6 x 150mm). TAGs were calculated as relative contents to the spiked d5-TAG 48:0 internal standard (CDN isotopes), while cholesteryl esters were normalized to corresponding d6-C18 cholesteryl ester (CDN isotopes). DAG species were quantified using 4ME 16:0 Diether DG as an internal standard (Avanti Polar Lipids, Alabaster, AL, USA). Free cholesterol was quantified using a HPLC atmospheric pressure chemical ionisation MS (HPLC/APCI/MS) method modified from a previously described protocol [8], with d6-cholesterol as an internal standard.

**Gas chromatography / mass spectrometry**

Aliquots of lipid extracts prepared as for LC/MS above were dried in a glass vial and 0.25 mL water was added followed by 0.25 mL 1 M KOH (in pure methanol). The vial was purged with argon gas and closed with a Teflon cap. After hydrolysis at 23°C for 16 h in the dark, the sample was acidified with 1.5 mL 80 mM acetic acid and 1.5 mL 0.1 M HCl. Solid-phase extraction was carried out on a 60 mg MAX (mixed ion exchange, Waters) column preconditioned with 2 mL methanol and then 2 mL 40 mM acetate buffer (pH 4.5). The extract was then loaded and the column was washed with 2 mL methanol / 20 mM formic acid, pH 4.5 (40/60), and subsequently dried for 2 min. Cholesterol and oxidized sterols were eluted with 2 mL hexane followed by 2 mL acetate/hexane (30/70). The eluted samples were evaporated under a stream of nitrogen and then derivatized with 25 µL acetonitrile and 25 µL of silylating agent N,O-bis(trimethylsilyl)trifluoroacetamide (BSTFA) containing 1% trimethylchlorosilane (TMCS) from Pierce Chemicals (Rockford, IL, USA) for 1 h at 22˚C.

Derivatized samples were analysed using an Agilent 5975 inert XL mass selective detector and 5973 gas chromatograph equipped with an automatic sampler and a computer workstation. Helium was used as the carrier gas at a flow rate of 0.8 mL/min. Derivatised samples (1 µL) were injected splitless into the GC injection port (280°C). Column temperature was increased from 160°C to 300°C at 40°C/min after 1 min at 160°C, then held at 300°C for 6 min. Selective ion monitoring was performed using electron ionization mode at 70eV (with ion source maintained at 230°C and the quadrupole at 150°C) to monitor one target ion and 2 qualifier ions selected from each compound’s mass spectrum to optimize sensitivity and specificity. Quantification of oxysterols was achieved by relating its peak area of target ion to its corresponding internal standard peak.

Desmosterol-d_6_, 14-demethyl-lanosterol-d_6_, zymosterol-d_5_, 14-demethyl-lanosterol, zymosterol and lanosterol were obtained from Avanti lipids and α-cholestane, squalene, 7α–hydroxycholesterol, 7β–hydroxycholesterol, 7-dehydrocholesterol, cholesterol 5 β-6-epoxide, cholesterol 5 α-6-epoxide, 25-hydroxycholesterol and 7-ketocholesterol were from Sigma (St. Louis, MO, USA). Lathosterol, desmosterol, β-sitosterol, campesterol, stigmasterol, 27-hydroxycholesterol were obtained from Steraloids (Newport, RI, USA). 7α-hydroxycholesterol-d_7_, 7β–hydroxycholesterol-d_7_, β-sitosterol-d_7_, campesterol-d_3_, lathosterol-d_4_ and 7-ketocholesterol-d_7_ were purchased from CDN Isotopes (Quebec, Canada); 27-hydroxycholesterol-d_5_, 24-hydroxycholesterol and 24-hydroxycholestero-d_7_ were from Medical Isotopes, Inc (Pelham, AL, USA). All standards obtained were of the highest purity (>95%).

**Electrospray ionisation MS**

Samples were homogenized in methanol:chloroform (2:1 v/v) containing 0.01 % butylated hydroxytoluene (BHT) at a ratio of 20:1 solvent to tissue. SM (d18:0/12:0) and Cer (d18:0/17:0) standards where added to the homogenates (4 μL of a 250 μM stock per mg of tissue). To improve the detection of SM, hydrolysis of glycerophospholipid fatty acyl esters was promoted by the addition of sodium hydroxide (0.7 M final concentration) to the homogenate prior to mixing in a tube rotator overnight at 4 °C [9]. Lipids were extracted by standard methods [10] with minor modifications [11]. Samples were stored at -80ºC until analysed. All mass spectra were obtained using a Waters QuattroMicro^TM^ (Waters, Manchester, U.K.) equipped with a z-spray electrospray ion source. Capillary voltage was set to 3000 V, source temperature 80 °C, desolvation temperature 120 °C. Cone voltage was set to 50 V and 35 V in negative and positive ion modes, respectively. Nitrogen was used as the drying gas at a flow rate of 320 L/h. Lipid extracts were diluted to approximately 40 μM with the addition of methanol:chloroform (2:1 v/v) and aqueous ammonium acetate (1M) at 50 µL/mL. Samples were infused into the electrospray ion source at a flow rate of 10 μL/min using the instrument’s on-board syringe pump and lipids quantified as previously described [11]. In all precursor ion, neutral loss and product ion scans, argon was used as the collision gas at a pressure of 3 mTorr. Sphingomyelins were analysed by performing precursor ion scans of *m/z* 184 (phosphocholine) in positive ion mode with a collision energy of 35 eV [12]. For ceramides, collision energy was set to 35 eV for negative ion mode neutral loss scans of d18:1 (256.3 Da) and d18:0 (258.3 Da) sphingoid bases [13]. Individual molecules were quantified by comparison to the appropriate internal standard after correction for isotope contributions as described previously [11].

**Variability associated with analysis of lipids by mass spectrometry**

Samples were all subjected to a single analysis using the different LC/MS, GC/MS and ESI/MS methods. The variability for replicate analysis of tissue samples using the NUS LC/MS and GC/MS methods has been previously determined to be 5% to 10% (Shui G, Wenk MR, unpublished data). The variability related to the UOW ESI/MS method has been previously determined to be 8% for the sphingolipid species analysed (Mitchell TW, unpublished data).

**High performance liquid chromatography**

Cholesterol and α-tocopherol content of brain tissue was determined by reversed-phase HPLC as described previously [14]. The cholesterol and α-tocopherol standards were purchased from Sigma (St Louis, MO, USA). Internal standard [3H]-cholesterol was purchased from GE Healthcare (Buckinghamshire, UK). In brief, 100 mg of tissue was homogenised in methanol:hexane (1:4, v:v) including 0.01% BHT and trace amounts of [3H]-cholesterol as an internal standard. Samples were placed in ice for 10 min, vortex mixed, and subsequently centrifuged at 3000 *g* for 15 min at 4°C. The hexane phase was collected and dried down under vacuum in a Speedvac centrifuge. Lipids were redissolved in 300 uL of isopropanol:acetonitrile (60:40, v:v) and 10 μL was assessed for [3H] by scintillation counting while 50 μL was injected onto a C18 reversed phase HPLC column as described [14].

**Quantitative real-time PCR**

Before RNA extraction, specificity of the primers was confirmed by demonstration of a single PCR product of the correct size as judged by agarose gel electrophoresis. All RNA procedures were carried out with RNase-free reagents. RNA was isolated from 50 mg brain tissue by homogenization in TRIzol reagent (Invitrogen, Mount Waverly, Australia) following the manufacturer’s protocol. RNA concentration was determined spectrophotometrically with Nanodrop 1000 (Thermo scientific, Wilmington, DE). Five μg of total RNA was used for reverse transcription with random primers and M-MLV reverse transcriptase (Promega, Sydney, Australia). The resulting cDNA provided the template in the qRT-PCR, which was carried out using a Mastercycler EP Realplex S (Eppendorf, North Ryde, Australia). Each reaction of 20 μL contained 12 μL RNase-free water, 5 μL IQ SYBR Green supermix (Biorad, Gladesville, Australia), 1 μL 5-20 pmol of primers, and 1μL of template. Amplification was carried out with 40 cycles of 94°C for 15 s and 60°C for 1 min. qRT-PCR of the house keeping gene, β-actin was also performed for each cDNA template and gene expression normalised to β-actin. RNA integrity was confirmed using a high resolution Bioanalyzer electrophoresis system (Agilent Technologies, Palo Alto, CA, USA) as described previously [15]. All primers were purchased from Sigma (Castle Hill, Australia) and details of the sequences are provided in Supporting Information Table S1. Primers were designed using Primer3 software (available at http://www.ncbi.nlm.nih.gov/) and based on the National Center for Biotechnology Information (NCBI) reference sequences. The specificity of the primers was confirmed by demonstration of a single PCR product of the correct size as judged by agarose gel electrophoresis. To validate the quality of the qRT-PCR measurements, melting curves were determined on amplification products generated from each cDNA template and, in all cases, a single melting temperature peak was observed indicating the presence of only one amplification product.

**References**

1. Elliott DA, Tsoi K, Holinkova S, Chan SL, Kim WS, et al. (2011) Isoform-specific proteolysis of apolipoprotein-E in the brain. Neurobiol Aging 32: 257-271.

2. Tong J, Wong H, Guttman M, Ang LC, Forno LS, et al. (2010) Brain alpha-synuclein accumulation in multiple system atrophy, Parkinson's disease and progressive supranuclear palsy: a comparative investigation. Brain 133: 172-188.

3. Bligh EG, Dyer WJ (1959) A rapid method of total lipid extraction and purification. Can J Biochem Physiol 37: 911-917.

4. Shui G, Guan XL, Gopalakrishnan P, Xue Y, Goh JS, et al. (2010) Characterization of substrate preference for Slc1p and Cst26p in Saccharomyces cerevisiae using lipidomic approaches and an LPAAT activity assay. PLoS ONE 5: e11956.

5. Chan R, Uchil PD, Jin J, Shui G, Ott DE, et al. (2008) Retroviruses human immunodeficiency virus and murine leukemia virus are enriched in phosphoinositides. J Virol 82: 11228-11238.

6. Shui G, Guan XL, Low CP, Chua GH, Goh JS, et al. (2010) Toward one step analysis of cellular lipidomes using liquid chromatography coupled with mass spectrometry: application to Saccharomyces cerevisiae and Schizosaccharomyces pombe lipidomics. Mol Biosyst 6: 1008-1017.

7. Oliveira TG, Chan RB, Tian H, Laredo M, Shui G, et al. (2010) Phospholipase D2 ablation ameliorates Alzheimer's disease-linked synaptic dysfunction and cognitive deficits. J Neurosci 30: 16419-16428.

8. Huang Q, Shen HM, Shui G, Wenk MR, Ong CN (2006) Emodin inhibits tumor cell adhesion through disruption of the membrane lipid Raft-associated integrin signaling pathway. Cancer Res 66: 5807-5815.

9. Le Lay S, Li Q, Proschogo N, Rodriguez M, Gunaratnam K, et al. (2009) Caveolin-1-dependent and -independent membrane domains. J Lipid Res 50: 1609-1620.

10. Folch J, Lees M, Sloane Stanley GH (1957) A simple method for the isolation and purifcation of total lipids from animal tissues. J Biol Chem 226: 497-501.

11. Deeley JM, Mitchell TW, Wei X, Korth J, Nealon JR, et al. (2008) Human lens lipids differ markedly from those of commonly used experimental animals. Biochim Biophys Acta 1781: 288-298.

12. Brugger B, Erben G, Sandhoff R, Wieland FT, Lehmann WD (1997) Quantitative analysis of biological membrane lipids at the low picomole level by nano-electrospray ionization tandem mass spectrometry. Proc Natl Acad Sci U S A 94: 2339-2344.

13. Han X (2002) Characterization and direct quantitation of ceramide molecular species from lipid extracts of biological samples by electrospray ionization tandem mass spectrometry. Anal Biochem 302: 199-212.

14. Fedorow H, Pickford R, Hook JM, Double KL, Halliday GM, et al. (2005) Dolichol is the major lipid component of human substantia nigra neuromelanin. J Neurochem 92: 990-995.

15. Kim WS, Wong J, Weickert CS, Webster MJ, Bahn S, et al. (2009) Apolipoprotein-D expression is increased during development and maturation of the human prefrontal cortex. J Neurochem 109: 1053-1066.
